# Development of a comprehensive school anti-bullying logic model in Abu Dhabi: a multi-method participatory approach

**DOI:** 10.3389/fpubh.2025.1649884

**Published:** 2025-08-20

**Authors:** Alfan Al-ketbi

**Affiliations:** Department of Health Care Management, College of Health Sciences, University of Sharjah, Sharjah, United Arab Emirates

**Keywords:** school-bullying, logic models, mental health, academic performance, UAE

## Abstract

**Background:**

Bullying remains a critical issue affecting the well-being, academic performance, and long-term development of school-aged children. This study documents the participatory development of a Comprehensive School Anti-Bullying Logic Model in the Emirate of Abu Dhabi, United Arab Emirates (UAE).

**Methods:**

Utilizing a multi-method process consisting of stakeholders’ interviews, focus group discussions (FGDs), group workshops, and a scoping review—the study sought to construct a culturally sensitive and actionable anti-bullying logic model.

**Results:**

Themes that emerged from interviews and FGDs were underreporting of bullying, the requirement of clearer policies, and a call for more support mechanisms. The logic model developed has organized components: inputs/resources, activities, outputs, outcomes, impact and assumption and external factors. This model combines global best practices with local knowledge and provides a strategic framework for efficient policy design and implementation for all schools in the Emirate. The process emphasizes the necessity of multi-stakeholder involvement in solving intricate education problems. This collaborative process led to a strategic, culturally suitable anti-bullying logic model. The model provides a blueprint for effective implementation and evaluation within and between schools in the emirate. The creation of this Abu Dhabi Comprehensive School Anti-Bullying Logic Model is a reflective and culturally appropriate approach in reducing bullying in schools. The utilization of a multi-method study and involving a broad cross-section of stakeholders has yielded a framework that is underpinned by local context and international best practice.

**Conclusion:**

This model offers a strategic roadmap for schools to effectively implement policies and programs to minimize bullying and establish a safer, more supportive learning environment. The research highlights the need for concerted effort in solving intricate problems such as bullying to ensure that solutions are practical, relevant, and sustainable.

## 1 Introduction

Bullying, defined as intentional, repeated aggressive behavior involving an imbalance of power, bullying can be physical, verbal, relational, or increasingly, digital (cyberbullying) in nature (1), is highly prevalent and variable in form (1–3). In terms of labeling behavior as bullying, repetition, intentionality, and a power imbalance are required conditions (2–4).

School bullying has been observed to happen in various cultures all over the world (5), where around one-third of children across the world go through bullying and school violence (6). Within the United Arab Emirates (UAE) context, research regarding bullying has begun to pick up pace, uncovering comparable issues (7). High number of students have been reported to be bullied (8, 9). Another Abu Dhabi school study emphasized that both students and employees frequently lacked the training and resources to spot, report, and intervene in bullying incidents (10). The results confirm regional concerns within the Gulf Cooperation Council (GCC) where youth mental health and school safety are becoming policy priorities (11).

In a bid to manage bullying among schoolchildren, various programs meant for bullying prevention have been designed and integrated into schools worldwide (12). School-based anti-bullying programs are the most common (8), where the aim is to equip children with resources and strategies to resist bullying (13–15). These anti-bullying programs are known by various names, e.g., bullying prevention programs (16), anti-bullying programs (17), social skills training programs (13, 18), and conflict resolution programs (19). Nonetheless, they involve an unstated assumption about the way bullying behavior in schools is to be handled. Some interventions aim to assist the victimized students and enable them to cope with bullying behaviors (20–22). Other interventions target modifying the school’s social climate and preventing all students’ behavior (19).

In recent years, great attention has been diverted to identify programs explicitly targeting bystander behavior in the school setting (23, 24). In an attempt to tackle the bullying problem comprehensively, logic models are normally formulated and applied (25). Application of Logic Models as an intentional prevention strategy in responding to numerous behavior problems in community-places, including school setting, have also been applied widely in context of youth engagement (26), prevention of sexual violence (27), and teen listener training (28). Logic models are practical and visual tools employed to plan interventions, develop plans for the evaluation of interventions, track programs of work, carry out research, and report the results of research (24, 29). The central emphasis of the application of logic models as a tool for assisting in design, planning, organization and representation of the outcome through the formulation and sharing of program theory (30). Program theory is an express theory of the manner in which an intervention causes a chain of processes and outcomes that can influence participants’ results and contribute to broader impact (31). A logic model is one program theory configuration, when applied enables researchers to articulate and explain how an intervention is postulated to effect outcomes and the main pathways and moderators to outcome achievement.

**Figure 1:**
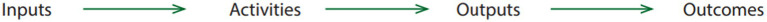
The simplest form of a logic model: inputs are the various resources available to support the program (e.g., staff, materials, curricula, funding, and equipment). Activities are the action components of the program (e.g., develop or select a curriculum, write a plan, implement a curriculum, train educators, pull together a coalition). These are sometimes referred to as process objectives. Outcomes are the intended accomplishments of the program. They include short-term, intermediate, and long-term or distal outcomes.

There are many ways to depict logic models as these may be simple or complex. The type and complexity of the logic model will depend on program focus, the purpose of the logic model, and the audience. Sometimes, programs may utilize several logic models with differing levels of complexity for different purposes and audiences or to highlight different program elements (32). The simplest form of a logic model includes four components as shown in Figure 1.

Logic models illustrate sequential chains of events or processes; and when interpreting, most logic models may be read as a series of “if … then ….” Statements (32, 33). The simple model outlined in the text gives a clear illustration of a cause (if)-and-effect (then) relationship in an intervention. This “if … then” structure basically describes the sequence of actions in the intervention, with each action precipitating an intended result. Collectively, these “if … then” statements describe a step-by-step logical process by which each action hinges on the other, establishing a chain of causality that results in the eventual outcome: prevention of diabetes exacerbations through improved education and drug compliance. Additionally, if, for instance, the logic model depicted in Figure 1 is dissected further, the “if … then” story can be constructed as below: “If teachers teach and a curriculum is developed on diabetes self-management, then more children are given diabetes self-management education.” This supposes that if a curriculum (particularly on diabetes self-management) is developed and taught by teachers, then more children will be given education on how to manage their diabetes. This is an underlying assumption, implying that the intervention will find its target group and the material will be well transmitted. “If children get diabetes self-management education, then their diabetes medication adherence improves.” The second step assumes that learning about diabetes management by children will result in better compliance with their asthma medication treatment. The reasoning behind this is that children are given the information of why and how they must take their medication, and this can have a positive effect on their behavior. Medication adherence and diabetes exacerbations will probably happen. “If adherence to diabetes medication improves, then diabetes exacerbations decrease.” The last link in the chain indicates that better adherence to medication will result in less fluctuation and abnormality in blood sugar levels. This presumes that adherence to medication protocols prescribed is central to the management of diabetes symptoms and avoiding exacerbations.

While many countries have constructed anti-bullying logic models, there is little evidence regarding the effectiveness and contextual suitability of these models in the United Arab Emirates (UAE), specifically Abu Dhabi. This research fills this void through the creation of a culturally responsive, integrated anti-bullying logic model for Abu Dhabi schools that addresses the unique needs and challenges of those schools. Interventions are scarce to address multiple types of school bullying in low- and middle-income countries, and few have been subject to rigorous testing (34–36). Exceptions are the “Good School Toolkit” (37, 38) in Uganda and the 130 sessions “Right to Play” Intervention, piloted in Grade 6 students in Pakistan (39).

The lack of effective interventions indicates the necessity of both further intervention development and piloting, as well as investigation of adaptation of highly successful interventions that already exist. Although earlier worldwide initiatives have generated useful models of bullying prevention (34, 35), most such frameworks have been developed without incorporating local stakeholders or tailored to the socio-cultural fabric of the UAE. For example, current Abu Dhabi school policies tend to be reactive in disciplinary measures rather than proactive systemic prevention approaches (40, 41). In addition, research shows that bullying in the area can be underreported as a result of stigma (42, 43), trust issues with reporting mechanisms (34, 39, 43, 44), and uncertain definitions of bullying actions (45)—factors typically not targeted in general interventions (46). Logic model strategy in healthcare research networks, this research transfers the same planning and evaluation rules to the education field (47).

Through the use of a participatory process among educators, students, parents, and policymakers, the research ensures that the outcome of the resulting logic model is not only evidence-based, but also locally grounded in real-world experience as well as cultural appropriateness. Drawing on a comprehensive literature review, the research team established that notwithstanding growing recognition of bullying in UAE schools, there are a number of fundamental gaps within the formulation and application of anti-bullying programs. To start with, most anti-bullying models brought into Abu Dhabi were borrowed from Western environments without adequate customization to native institutional arrangements, social patterns, and cultural practices (10). Such has led to interventions that may not fully represent the nature of Emirati and expatriate students. Most of these programs have been designed and delivered from a top-down perspective with minimal input from key stakeholders including the students, teachers, school counselors, and parents. Lacking participatory design makes the interventions to lack ownership in communities as well as non-sustainable. Third, the UAE’s education system has no established assessment tools to monitor, evaluate, and adjust anti-bullying programs over the years, which makes it impossible to measure the impact or replicate effectiveness (48). Lastly, whereas in other countries, logic models have been employed to inform program development and evaluation in education and public health, their application in school bullying prevention in the UAE is limited and underreported in the literature (49).

In order to meet the main gaps determined in the study, a number of main questions were formulated to inform the development of a successful anti-bullying intervention for Abu Dhabi schools. The research first queried what the key components of a robust anti-bullying logic model need to include—namely, the inputs, activities, outputs, outcomes, and final impact best suited for the immediate school environment. Second, it looked at how various groups—students, teachers, parents, and school administrators—view existing anti-bullying work and what they recommend as improvements. Third, the study investigated how employing participatory design practices, in which stakeholders are engaged directly in defining the program, may contribute to the development of a locally relevant, culturally appropriate, and sustained solution. Lastly, the research was meant to determine what short- and long-term alterations would be anticipated when such a logic model is completely brought to application in schools throughout Abu Dhabi.

Based on these research gaps and research questions, the primary aim of the current study is thus to create a culturally grounded school anti-bullying logic model through a participatory, multi-method process entailing qualitative research and professional consultation. The goal centers on crafting an organized framework that captures the distinct cultural, educational, and social features of Abu Dhabi schools. Realizing that most global anti-bullying models could not possibly conform to all local norms, values, and structures, the research utilized a participatory development process to provide contextual focus. The process integrated several qualitative research approaches, such as: (1) focus group discussions (FGDs) among students, parents, and teachers on experiencing and understanding lived lives, perceptions of bullying, and expectations of effective prevention; (2) Semi-structured interviews with school counselors and educational leaders on institutional challenges and system-level gaps; and (3) scoping review of international anti-bullying logic models and interventions to determine best practices adaptable and core components. The evolution of the logic model was also enhanced through expert panel sessions, in which education, child psychology, school health, and UAE school system experts reviewed and tested every element of the model. The experts gave systematic feedback on the model’s inputs (training, resources, policies), activities (school culture interventions, AI-driven reporting, awareness campaigns), short-term and long-term outcomes (behavioral change, decreased bullying), and facilitating contextual factors. This participatory, iterative process ensured that the resulting logic model was not only evidence-informed but also grounded in the cultural realities and operational dynamics of Abu Dhabi’s school system. The final model is designed to serve as a practical planning, implementation, and evaluation tool for bullying prevention efforts that are both effective and culturally appropriate.

## 2 Materials and methods

### 2.1 Research design

Mouton (50) offers a typology of research design to guide planning for a research study. Non-empirical research approach is adopted in this study. Mainly, this study is non-empirical in nature because it aims to examine, monitor and analyze current literature on school bullying. This study subsequently synthesizes literature review to systematically develop the Anti-Bullying logic model. Following this, an empirical research approach is conducted to gain knowledge and insight into the application of policy analysis on schools anti-bullying process. The outcomes presented by the logic model are subsequently used to present research findings on how policy analysis can be utilized as a decision-making model for an initiative such as schools Anti-Bullying initiative.

### 2.2 Research settings

School bullying is very common in UAE. Therefore, school bullying must be addressed by the UAE not only for the safety and well-being of students but also for the creation of a positive, inclusive, and successful learning environment that is consistent with national values and objectives. Preventing bullying helps to build a healthier, more resilient community, decreases mental health issues, improves academic success, and facilitates the development of responsible, empathetic citizens who will help make the UAE a prosperous and harmonious nation for generations to come. The scholars at the Institute of Public Health, College of Medicine and Health Sciences, United Arab Emirates University (UAEU) for the doctoral research on “Policymaker’s Perspectives and Student Experiences of Bullying at Schools in the UAE: A Mixed-Method Approach,” have already put out some pioneering work on the topic (51). From their work in this challenging but emerging health field, the group, as an initial starting point, has been engaged in developing a School Anti-Bullying Logic Model in Abu Dhabi as a potential solution and path forward for the prevalence of school bullying across the UAE.

### 2.3 Research participants

The participants (*n* = 12) comprised experts from public health, child protection center, UNICEF, telecommunications, Islamic affairs, statistics center, Ministry of Education (MOE), transport, judicial department, school health services and other related authorities and institutions. Participants in the interviews were recruited using purposive sampling and the snowball technique which was considered based on participant introduction of another participant to the study. Purposeful selection was utilized to choose staff for the interviews. Proper sample size in qualitative research, especially the number of interviews, is usually established when saturation is achieved. Sadly, little consensus exists about the definition of saturation, how to tell when it has been achieved, or whether this can be pre-determined in qualitative research. The sample size for this research was derived from the differential experience of the participants with bullying and the organizational position they held. This method aimed at gaining a well-rounded perspective of bullying (52). In this research, inclusion criteria of being older than 18 years and employed in the emirate of Abu Dhabi and exclusion criteria of being younger than 18 and residents of other emirates, were used. Participants characteristics are provided in Table 1.

**Table 1:** Participants characteristics – education and professional experience.

Code	Gender	Age	Level of education	Profession	Organization	Organization type	Years of experience
NA-UAEU	Female	45	PHD	Dean/Administrator	UAEU	Federal	
SA-AWQAF	Female		Bachelor	Religious Figure	AWQAF	Federal	
AA-TRA	Male	28	Bachelor	Security Analyst	TRA	Federal	3
AA-MOI	Male			Military Officer	MOI-CPC	Federal	10
IA-PMO	Male	54	PHD	Advisor	MOI-Aqder	Federal	54
MA-MOE	Female			Education Specialist	MOE	Federal	
FA-PMO	Female	28		Research Analyst	PMO	Federal	
NS-ADSHS	Female	50	Bachelor	Education Specialist	ADSHS	Local	17
SA-ED	Male	35		Trainer/Educator	ED	Private	14
OF-ET	Male	39		Safety Officer	ET	Federal	16
MA-SC	Male	39		Statistician	ADSC	Local	12
KK-MHC	Male	47		Child Mental Health Consultant	MHC	Private	20
ME-UNICEF	Male	64		Program Officer	UNICEF	International	6
MA-ADPHC	Female			Maternal and child health officer	ADPHC	Local	6
AA-ADDFP	Female			Prosecutor	ADDFP	Local	13

### 2.4 Data collection tools and procedure

Numerous primary data sources informed data collection. These included training on logic model, documents review, naturalistic observations, email correspondence and teleconferences, Focus Group Discussions (FGDs); structured Interviews and workshops.

#### 2.4.1 Logic model training sessions

The Research team organized a logic modeling training session and the related materials in hard and soft forms. The training utilized published reports on theory-driven, realist evaluation principles and logic modeling; and concentrated on how mapping program inputs, activities and outcomes can assist in developing early hypotheses for provisional relevant Contexts, Mechanisms, Outcomes and configurations. While a range of logic model formats were discussed during training, a columnar and multilayer format was selected to enable the outputs, activities, inputs, and outcomes to be identified. Possible contextual factors influencing the program outcomes and outputs were also identified and clarified by the participants. The identification process of contextual factors began by gathering a wide list of possible contextual factors that might affect processes required to attain inputs, outputs and outcomes in the antibullying program Team agreement was realized in accordance with the significance of the evaluation questions, academic rigor and the assumed role in possible outcome patterns. Contextual factors were subsequently ordered at micro, meso and macro levels utilizing knowledge from the conceptual framework for the realist evaluation.

#### 2.4.2 Document review

A checklist based on case study literature was used to list the common documents the research team were looking for. The kinds of documents they wanted to review were strategic plans/planning documents, operating procedures and organizational policies, communications (e.g., website, media releases, email, meeting minutes), annual reports, administrative databases/files, evaluation reports, third party consultant reports, and routine numerically collected data that captured implementation outcomes. As each document was found, it was dated, numbered, and listed in the case study database with a brief description of the content pertaining to the research objectives. A total of 57 documents were examined across the case sites.

#### 2.4.3 Naturalistic observations

The onsite observations were made over a period of 1 week, where normal organizational activities were observed. The research staff engaged school-staff, inquired, and clarified the observation that was occurring; they did not interfere with normal working practices. Observation enabled us to see how any antibullying program functioned and compare that with written processes and procedures. It also enabled the observation of non-verbal responses and staff interaction. 105 h were spent on the observations.

#### 2.4.4 Email correspondence and teleconferences

based on the above steps, a draft logic model was developed by the first author and was circulated with policy makers and implementers to set out their version of the model. In particular, monthly teleconferences and regular email discussions with implementers and policy makers helped to iterate and improve the draft model until it captured stakeholders’ collective thinking about how the anti-bullying program ought to function in the UAE context. Improving the logic model involved making explicit and agreeing on relationships between program components. Consultation and interaction with local stakeholders ensured that solely integral elements of the multi-intervention antibullying program were captured in the LM. The selection process of information to incorporate into the LM was iterative and dynamic, and entailed the addition of newly emerging/relevant components as well as the elimination of those considered unnecessary for responding to our evaluation questions.

Focus group discussions (FGDs), interviews and technical workshop: as a final step to further build consensus on the developed logic model, FDGs, interviews and technical workshops were used. Three focus group discussions (FGDs) were conducted in May 2021 with a small group of six to eight participants of the same experience and background in each FGD to discuss their interests and experiences about bullying. With the facilitation of these FGDs, information about what different groups of stakeholders and members of educational administration believe about the current bullying situation in Abu Dhabi, its interventions and implementation was collected. The duration of each FGD was 90 to 120 min and all three FGDs were recorded with a recorder and were transcribed in MS Word in Arabic and English. Individual interview participants were asked to take part in the study using purposive sampling in which participant referrals adopted the snowball technique. The interview participants inclusion criteria were to be above 18 years old and work within the emirate of Abu Dhabi, while those above 18 years old were excluded and others from other emirates. FDGs and interviewing were employed to disentangle program elements and contextual factors influencing the implementation and effects of antibullying program, and then formulate early working theories regarding how, why and in what contexts the theories can function.

### 2.5 Checklist to evaluate the proposed model

The final model was assessed by a group of experts (*n* = 5) against a checklist prepared for the purpose of the present study using criteria set by some previous work (53, 54). These experts provided a written consent form prior to participate in the study. The experts used this checklist with questions on different parts of the logic model. The experts were asked to respond each question with notes, if applicable, and write in front of each question “yes/no/”. the experts also provided general comments, in the light of which, the model was refined. A summary of these comments is provided in Supplementary File S1.

### 2.6 Data analysis

Data collected from various sources—document reviews, stakeholder consultations, observations, interviews, and focus group discussion—were analyzed with a mix of thematic content analysis and realist evaluation-informed framework analysis. FGDs and interviews were audio-recorded, transcribed, and inductively coded to reveal patterns and emerging themes on bullying perceptions, implementation challenges, contextual factors, and hoped-for outcomes. Applying the Iterative Logic Model (ILM) framework, qualitative data were used to map against key logic model constructs: inputs, activities, outputs, outcomes, and contextual factors at micro, meso, and macro levels. This mapping facilitated the building and refinement of an extensive anti-bullying logic model. Findings were triangulated and validated through training session, teleconference, and document review data. The analysis was done in collaboration, with ongoing consultations involving stakeholders to achieve credibility, relevance, and contextual fit of the end model. NVivo or equivalent qualitative software was used optionally to handle excessive qualitative data and ensure coding consistency across the researchers.

The study was approved from the UAE University Human Medical Research Ethics Committee (ERS_2019_5867).

## 3 Results

**Figure 2:**
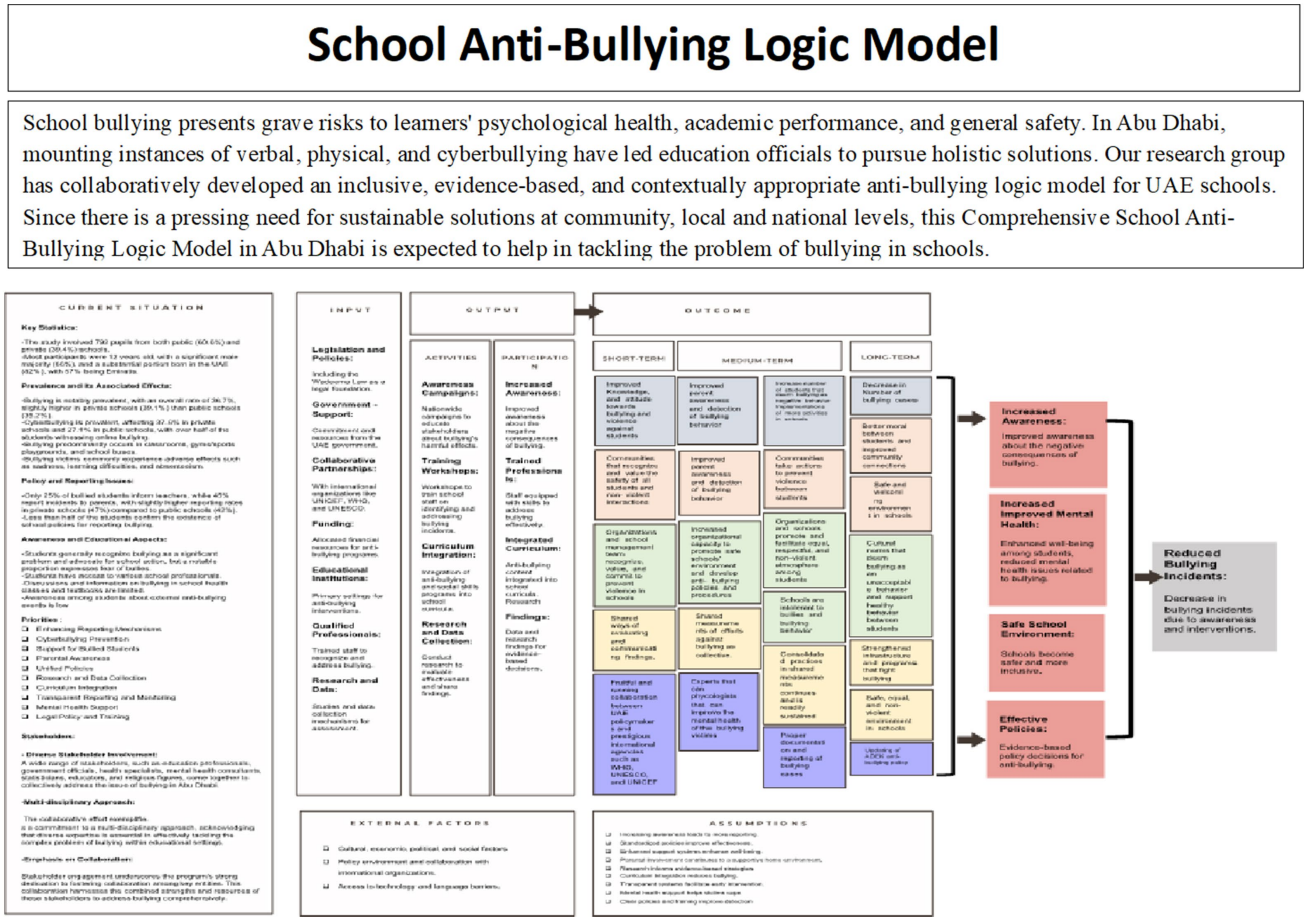
The UAE schools anti-bullying logic model.

The logic model developed in this study is illustrated in Figure 2. Key components of the Logic Model are given in Table 1. Further details on UAE Schools Antibullying Logic Model are provided in Supplementary Tables 1–6.

This research yielded the creation of a Comprehensive School Anti-Bullying Logic Model that was tailor-made for Abu Dhabi schools. Empirically informed and stakeholder-inclusive in nature, the model presents a strategic blueprint for countering bullying within UAE school systems. The logic model is composed of five interconnected elements: Inputs, Activities and Outputs, Outcomes (Short-, Medium-, and Long-Term), and Assumptions and External Factors.

### 3.1 Current situation and problem identification

The model was created as a reaction to widespread bullying problems realized in the local setting. Whereas more than 75% of students in public and private schools have reportedly been bullied, and low rates of incident reporting, there is a great need for a systemic, culturally specific intervention. Current anti-bullying programs are plagued by variable implementation, inadequate stakeholder engagement, and absence of monitoring and evaluation mechanisms.

### 3.2 Inputs

The model specifies core resources and institutional processes required to implement anti-bullying interventions including:

a) Legislation and Policy: Harmonization with UAE legislations and child protection laws.
b) Governmental Commitment: Endorsement and strategic alignment from the Ministry of Education and concerned authorities.
c) Partnerships: Active collaboration with partners like WHO, UNICEF, school boards, judicial agencies, and healthcare services.
Educational Institutions: Engagement of schools and universities as implementation and research partners.
d) Funding and Resources: Financial and technical assistance for training, AI-powered reporting systems, awareness campaigns, and program assessment.

### 3.3 Outputs (activities and participation)

Major activities undertaken to achieve the model goals were:

a) Awareness Campaigns: Grassroots activity to create awareness regarding bullying and its effects.
b) Training Workshops: Capacity development for teachers, counselors, and administrative staff.
c) Curriculum Integration: Incorporating anti-bullying messages in academic and civic education.
d) Research and Data Collection: Collection of qualitative and quantitative data to aid in model validation and refinement.
e) Participation of Stakeholders: Participative inclusion of teachers, students, parents, policy-makers, and outside experts.
f) These outputs combined were intended to produce trained professionals, enhanced curriculum content, and participative stakeholders able to identify and act against bullying.

### 3.4 Outcomes

A. Short-Term Outcomes:

a) Increased awareness among students and staff about bullying behaviors and preventive measures.
b) Greater awareness of support mechanisms and reporting routes.
c) Common conceptualization of intervention objectives and program logic across stakeholders.
d) Early institutional buy-in to adopt anti-bullying efforts.

B. Medium-Term Outcomes:

a) Relatable improvements in student conduct and peer interactions.
b) Improved coordination among education, health, and judiciary sectors.
c) Incorporation of welcoming school policies and provision of mental health services.
d) Sufficient early evidence in favor of replication and scaling of the intervention to other emirates.

C. Long-Term Outcomes:

a) Drastic decrease in reported bullying incidents.
b) Improved student mental well-being, academic achievement, and school participation.
c) Development of safe and welcoming learning spaces.
d) Institutionalization of evidence-based anti-bullying policies nationally.

### 3.5 Assumptions and external factors

The logic model is based on assumptions such as sustained stakeholder participation, cultural awareness, sustainable funding, and technology integration. External influences like social norms, political processes, media impact, and language differences were also identified as potential impinging factors for the success of the model.

### 3.6 Impact pathway

Through a coherent and evidence-based sequence of inputs, activities, and outcomes, the logic model demonstrates a viable pathway to reducing bullying incidents. The model integrates improved awareness, mental health support, policy reform, and safer school environments, contributing to a more holistic and sustainable solution to school-based bullying in Abu Dhabi.

### 3.7 Checklist used for evaluation of the logic model

The results on the checklist used for evaluation of the logic models demonstrate that,

1. The structure of the logic model is strong and adheres to all Global Affairs standards. It is coherent, horizontally and vertically logical, and clear to external assessors.
2. The ultimate outcome addresses all criteria and is logically connected to lower-level changes and the theory of change for the project. The intermediate outcomes are logical, measurable, achievable, and connected to the ultimate outcome with complete regard for equity and human rights.
3. The immediate outcomes in the logic model represent focused capacity changes, are logically connected to outputs and intermediate outcomes, and both measurable and attainable within the project’s scope.
4. The outputs in the model are specific, properly constituted, and directly connected to the immediate outcomes. They are measurable, evidence-based, and sensibly posited within the scope of the anti-bullying project.
5. The theory-of-change story (based on the assumed anti-bullying model) is exhaustive and satisfies all evaluation requirements. It links logic model components transparently, addresses inclusivity and human rights, foresees risks, and exhibits systemic thinking.

A summary of the checklist evaluation is provided in Table 2.

**Table 2:** Summary of the checklist to evaluate the proposed anti-bullying logic model.

S. No.	Criterion	Met?	Notes
1	Uses internationally recognized and/or Global Affairs Canada logic model template	Yes	Clearly follows required structure
2	One outcome per box	Yes	No compound or multiple ideas per outcome
3	One ultimate outcome	Yes	Focused on student well-being
4	2–3 intermediate outcomes	Yes	Three defined
5	2 immediate outcomes per intermediate outcome	Yes	Logical and achievable links
6	1–3 outputs per immediate outcome	Yes	Relevant and measurable
7	Outputs placed beneath immediate outcomes	Yes	Proper structure followed
8	Fits on one page	Yes	Concise and visually clear
9	Logical vertical relationships	Yes	Clear cause-effect chain from outputs to outcomes
10	Logical horizontal complementarity	Yes	Each outcome is distinct yet contributes to higher-level change
11	Presents an evidence-based theory of change	Yes	Informed by stakeholder inputs and a scoping review
12	Addresses the original problem	Yes	Focuses on bullying reduction in schools
13	Aligns with regional/country program outcomes	Yes	Linked to education and child protection goals
14	Understandable to external audiences	Yes	Clear, jargon-free statements

In summary, the logic model developed for the anti-bullying intervention in Abu Dhabi schools adheres closely to the structural standards established by Global Affairs Canada. The model includes a single, clearly defined ultimate outcome supported by a realistic number of intermediate outcomes, each of which is logically linked to two immediate outcomes and associated outputs, ensuring vertical alignment and traceability of results. Each is typed into a separate cell in brief, one unique alteration to each statement, and eschews phrasing that is compound. The structure of the logic model is equally balanced, holds on one page, and outlines a vertically logical and horizontally complementary plan of the project’s theory of change. Outputs are clearly positioned under their associated immediate outcomes, and the logic model provides sufficient clarity for an external reviewer to comprehend the project’s projected changes and how they will be realized. Additionally, the model is consistent with wider programmatic priorities and demonstrates gender equality, human rights, and stakeholder engagement throughout. This format is a transparent, evidence-based framework that operationalizes the project’s theory of change and facilitates effective performance measurement.

## 4 Discussion

School bullying presents grave risks to learners’ psychological health, academic performance, and general safety. In Abu Dhabi, mounting instances of verbal, physical, and cyberbullying have led education officials to pursue holistic solutions (55). Acutely aware of the limitations of disjointed or responsive interventions, this research sought to collaboratively develop an inclusive, evidence-based, and contextually appropriate anti-bullying logic model for UAE schools. Since there is a pressing need for sustainable solutions at community, local and national levels, the current study is an important attempt to formulate a Comprehensive School Anti-Bullying Logic Model in Abu Dhabi to tackle the problem of bullying in schools.

This study also used a systematic method grounded on Global Affairs Canada’s Results-Based Management (RBM) framework to design and test the logic model underpinning the Abu Dhabi school anti-bullying intervention (53). The RBM framework offers a systematic means of describing, monitoring, and measuring development programs through clear-cut outcomes at multiple levels—final, intermediate, immediate, and outputs. The process was initiated with a well-defined mission: establishing a secure and protective school climate in Abu Dhabi through the systematic reduction of bullying. The intended audience was students, educators, parents, school personnel, and policymakers. In line with Hayes et al. stakeholder involvement was a key factor (47). Adopting a participatory approach akin to the PBRN method, stakeholders were meaningfully engaged via interviews, focus group discussions, and workshops. This served to ensure the logic model captured different viewpoints and local applicability. The process developed through a coherent sequence that started with knowing the wider context, such as institutional objectives, current initiatives, and contextual issues. This guided the priorities and objectives of the program. Inputs were identified as resources such as trained personnel, finance, educational resources, and technology. Activities comprised campaigns, training teachers, counseling students, and policy formulation. Outputs were concrete and quantifiable, such as the number of workshops organized or materials handed out. Process indicators and strategies were employed to assess if intended activity had been implemented efficiently and reached the target population. Outcomes were measured in short- to medium-term terms, for example, enhanced awareness and better reporting of bullying. Indicators supported measuring progress toward these goals. Lastly, long-term effects such as decreases in bullying and the establishment of more secure school settings were tracked to assess the overall effectiveness of the intervention.

Development of this model was partly guided by the process described by Hayes et al., for their article on logic models for evaluation and planning in primary care practice-based research networks (PBRNs) (47). They provide a focus on an organized process involving mission statement development, stakeholder engagement, and explicit identification of inputs, activities, outputs, outcomes, and impact.

In addition, this research also partly used a systematic method using the Global Affairs Canada. Results-Based Management (RBM) (53) approach to develop and evaluate the logic model that supports the Abu Dhabi school anti-bullying intervention. The RBM system provides a systematic means of specifying, tracking, and judging development interventions by concentrating on well-stated results at several levels: ultimate result, intermediate results, immediate results, and outputs.

**Figure 3:**
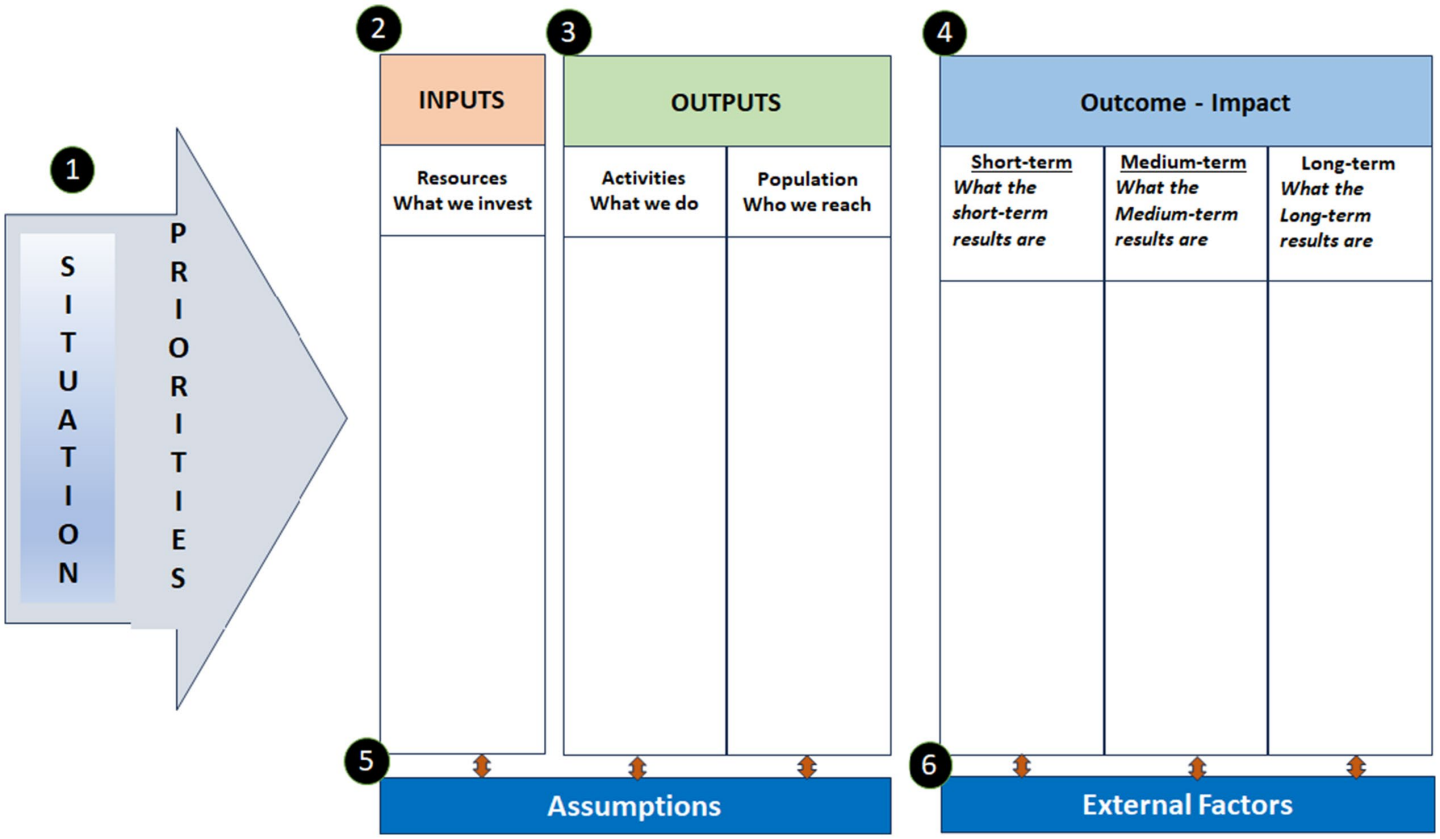
The logic model template (28).

Once a rough draft had been established (Figure 3), it was useful to specify and recognize assumptions and external factors. Assumptions were defined as conditions regarding how the program will function and the individuals that are involved. Based on assumptions regarding the external and internal environment, what the program hopes to accomplish, and the participants learning styles, behavior, and motivation. It’s necessary to say assumptions out loud as a group in order to recognize and change faulty assumptions. External Factors included the environment or context in which the program or project was found to occur (e.g., schools). External factors consisted of: the culture, the weather, economic structure, housing patterns, demographic patterns, political climate, participants’ background and experiences, media influence, and shifting policies. It is worth taking into consideration the hindrances that these factors bring but leveraging them as strengths to enable advancement.

The methodology is participatory since it engaged multiple stakeholders in the model development and design, such that the resulting product was not only useful but also appropriate for the cultural and local traditions and culture of UAE. The study incorporated a multi-method strategy, which enabled deep investigation into the problem and the development of a strong framework. A Multi-Method Approach was utilized with stakeholder interviews, focus group discussions (FGDs), group workshops, and scoping review used as the strategies for gathering the insights and developing the Logic Model (40). This triangulation of the qualitative and review methods enabled the researchers to conduct data triangulation to achieve an in-depth grasp of the problem of bullying and how it would be solved. Stakeholder Interviews were performed with essential individuals, like educators, administrators, parents, and students, to collect detailed personal views on the existing situation of bullying in schools. Interviews possibly uncovered loopholes in present policies, practices, and resources. Focus Group Discussions (FGDs) provided participants with the opportunity to engage in a more interactive discussion about bullying, allowing researchers to identify common issues, shared experiences, and solutions. FGDs would have offered some insight into perceptions about bullying across various community members and offered a greater understanding of local issues and expectations. These workshops included stakeholders working together to generate ideas and solutions. They probably allowed participants to work on hands-on problem-solving, providing tangible and actionable recommendations for anti-bullying interventions. A scoping review is a research technique applied to map out current literature on a given topic. In this instance, it entailed examining international best practices and current anti-bullying models from across the globe, which guided the creation of the Abu Dhabi-specific logic model. The interviews and FGDs identified several main themes that required mention in the logic model (51).

One of the major challenges discovered was bullying wasn’t being reported frequently enough. This may be due to fear of reprisal, unfamiliarity with the reporting procedures for bullying, or mistrust in the system. The logic model most probably answers this by recommending measures to facilitate the reporting procedures and build the system’s credibility. There was a perceived demand for more specific and transparent anti-bullying policies (56). Clear, consistent policies would assist in ensuring all parties involved—students, teachers, parents, and administrators—knew what bullying was and what should be done if and when it happened. Our study suggested that further support structures be implemented to guide students who are victims of bullying and students with bullying tendencies (57). These structures would encompass counseling sessions (58), peer counseling groups (59), and staff development for educators on how to effectively manage bullying (60).

The research generated a Comprehensive School Anti-Bullying Logic Model with five organized components:

*1. Situation and Priorities*: The situational factor with the greatest influence on the focus and priorities of the program is the rising trend and patterns of school-bullying in UAE, which has been on the rise for years (55). more worrisome is the fact that despite countless efforts made in every corner of this problem in school bullying at several levels to lock down, still it feels that the phenomenon has a tendency of escalating toward being more commonplace. Studies have demonstrated that multisectoral strategy with one objective and clearly defined aims is the key to solving the problem since school bulling is a multifaced issue and solving it must be based on the involvement of all the stakeholders (61).
*2. Inputs/Resources*: This is the resources needed to put the anti-bullying program into practice effectively. These may be funding, trained personnel, educational program materials, or outside expertise. What activities we do. These are the interventions or actions that schools will take to prevent and deal with bullying. Activities may include anti-bullying campaigns, staff and student training, support network establishment, and reporting systems development. What population we reach. The primary subjects are those inside the school environment (students, teachers, administration) and outside the school environment (local community, administration, parents etc.).
*3. Outcomes*: Outputs are the tangible outcomes of the activities, including the number trained students, bullying incidents reported numbers, or providing support services for schools. They assist in capturing the intervention’s immediate effects. These may also be the longer-term consequences that arise due to the activities and outputs. For instance, better student well-being, higher awareness and knowledge about bullying, and a reduction in bullying incidents are likely desired outcomes.
*4. Impact*: The final objective of the logic model is to have a sustained impact, i.e., a cultural change in the perception and management of bullying within schools and a decrease in overall bullying activity throughout the Emirate.
*5. Assumptions*: certain assumptions were made in the present model developed in this study. For example, “teachers and students will engage in and commit to the anti-bullying initiatives.”
*6. External Factors*: the external factors included in the model developed include, for example, cultural attitude, government policies, economic constraints, technological influences, etc.
*7. Evaluation:* This research investigation seeks to formulate a logic model that can help the education staff at school level, education authorities and government officials formulate policy-specific plans (via policy analysis) which will facilitate the operationalization of anti-bullying operating model from a policy viewpoint. Within this research, a suggested policy analysis approach is formulated, i.e., the anti-bullying logic model. For the purpose of demonstrating the correctness and sufficiency of the anti-bullying logic model logic model, the research findings that inform the development, including the anti-bullying logic model and its purpose need to be assessed. Assessment is needed to offer a verdict of the research findings and suggested anti-bullying logic model. In this research, the assessment process was split into two broad categories, verification and validation, that can be undertaken through four sequential stages: (i) theoretical verification; subject matter expert (SME) validation; (iii) case study validation; and (iv) transferability (62). Each step in the evaluation process is designed to build incrementally on the reliability and validity of the constructed anti bullying logic model. Each step has an input type—the method used to conduct the evaluation in each step. Each step also has one or two evaluation objectives to meet during the evaluation process. For the present work, however, only the first two of the four sequential stages were used as the last two were beyond the scope of this study. The developed anti-bullying logic model underwent a thorough verification process. This process includes input from Subject-Matter Experts (SMEs) with the relevant experience in and knowledge of the respective research areas, in order to assure that the anti-bullying logic model is developed based on valid, accurate and credible literature information and assumptions. The inputs that were placed into constructing the anti-bullying logic model gives confidence that the anti-bullying logic model has been “constructed right” (63). Once the inputs from the verification stage were integrated, the anti-bullying logic model was suggested to pertinent anti bullying specialists—asking for their professional feedback and certification to confirm that the anti-bullying logic model is applicable and fitting to the school bullying context and the conditions of this research. The validation by SMEs takes advantage of standardized questionnaires and semi-structured interviews (a workshop format) to obtain the opinion of experts on the anti-bullying logic model under development in this research. The SMEs give their inputs based solely on their knowledge and experience; thus, for assurance that the method of policy analysis can fulfill its intended use, a second validation strategy is explored to also test the usability of the anti-bullying logic model.

The present model developed in this study does not just represent international practices; it integrates them with local insights gained from stakeholders in Abu Dhabi. This is crucial because while international best practices offer valuable guidance, each community has unique needs, values, and challenges. By incorporating local perspectives (64), the model ensures that it is contextually relevant and more likely to be effective in Abu Dhabi’s schools.

One of the most important lessons to be learned from this research is the need for multi-stakeholder involvement (65). Bullying is a multifaceted problem that involves more than one stakeholder, including students, parents, teachers, and school administrators. Solving it needs feedback from all these stakeholders to determine the entire magnitude of the problem and to plan interventions that will be supported and executed effectively. The participatory aspect of the process also generates a sense of ownership and cooperation between stakeholders, which is vital for the sustainability of any anti-bullying program. This research suggests, however, that the future research should also consider incorporating the stakeholders mapping in applying anti-bullying policies within the school setting (65, 66).

The logic model of the current study is likely to be an active tool for planning, implementation, and evaluation. It helped in balancing activities with the outcome in mind and offered a guideline for constant assessment and improvement, which reflected the utility evidenced in PBRN environments. Through the application of the logic model framework used in primary care research to the Abu Dhabi school setting, a wide-ranging and culturally responsive strategy for addressing school bullying was created. This model not only informs ongoing interventions but also provides a basis for future evaluation and ongoing improvement (47). Logic models are very variable and adaptable: they may represent systems or processes, intervention or naturally occurring phenomena, or simple or intricate relationships (67). There are no specified criteria as to what is and what is not a logic model. Apart from this flexibility, all logic models have the characteristic of being: (i) visual theories; that (ii) depict not only concepts, but the concepts’ relationship as well; and that (iii) are formed based on a clear set of explanations regarding how/why relationship happens between concepts founded on sound theories, evidence, and/or professional and experience-based expertise.

Our research team performed a scoping review to gather best practices and frameworks internationally (40). These sources included UNESCO, WHO, and national anti-bullying strategies. Best models usually had elements such as awareness programs, employee training, reporting mechanisms, counseling services, and incorporation into policies. This scoping review is an analysis of school bullying intervention and prevention measures in the UAE and found 22 initiatives from both federal and private sectors from 2010 to 2021. It points out increased bullying, no peer-reviewed assessments, and sparse attention toward cyberbullying. The authors urge more robust studies, multisector collaborations, and evidence-based strategies to enhance national anti-bullying measures.

Cultural flexibility and stakeholder acceptance were found to be critical to success (49, 68). For the current logic model, five semi-structured interviews were done with principals, educators, policymakers, and child protection officers. Themes that emerged were policy inconsistency, underreporting, and resource constraints. Six FGDs were conducted with students, parents, teachers, and counselors. Students stressed anonymity in reporting; parents demanded involvement; teachers asked for training and materials. Besides, four workshops involving 35 stakeholders strengthened and cross-checked the logic model. Prioritization and consensus-building methodologies confirmed relevance and practicability. The evolution of the Comprehensive School Anti-Bullying Logic Model in Abu Dhabi illustrates how an organized, stakeholder-based approach can give rise to the implementation of a real-world, dynamic framework to confront intricate social concerns in schools. Using Hayes et al., logic model framework as the basis, this study was able to successfully transplant principles classically employed in primary care research networks (PBRNs) to an educational environment—in testament to the model’s strength across fields (47). One of the greatest strengths of this process was its participatory nature (47, 69). Similarly, Hayes et al. stressed the need to involve practice-based stakeholders in order to achieve relevance and sustainability in PBRNs. In this study, school administrators, teachers, parents, students, and policymakers were involved at all stages of the development process. Their input not only confirmed the elements of the model but also influenced its design to suit the cultural and institutional context of Abu Dhabi schools (47).

The sharp distinction between inputs, activities, outputs, outcomes, and impact facilitated the matching of short-term interventions with long-term objectives. The matching ensures that interventions like awareness campaigns, training for teachers, and counseling for students are not viewed as discrete actions but as part of an integrated system to bring about less bullying and better school culture (70).

The logic model developed in this study (Figure 2) offers a common language and an open framework for planning, executing, and assessing anti-bullying initiatives, an advantage also noted in Hayes et al.’s use of logic models in PBRNs (47). Additionally, the iterative and reflective model-building process reflected the ongoing learning cycle articulated in the PBRN logic model framework. Through repeated visits and model refinement through workshop and validation sessions, stakeholders-built buy-in and enhanced the potential for continued use at policy and practice levels. There were challenges, nonetheless. As with PBRNs, where diverse health professionals may interpret goals differently, educational stakeholders also exhibited varying understandings of bullying, especially concerning cultural sensitivity, cyberbullying, and gender dynamics. These discrepancies underline the need for ongoing training and consensus-building mechanisms even after the model is implemented (71, 72).

Lastly, the adaptability of the logic model suggests potential for wider application to the rest of the UAE and other education systems confronting similar challenges. Future studies should concentrate on the application of the model in real time in schools, measuring reductions in bullying prevalence, reporting behavior, and student well-being, to better refine and scale the intervention. Assumptions and external influences are essential components in a logic model that serve to clarify the context, boundaries, and possible issues of the intervention being undertaken (33). By knowing these elements, one can implement the model more realistically and successfully, as they define elements that may affect the success of the intervention but might not always be immediately under the control of the involved stakeholders.

The logic model for this study has also made assumptions that refer to the beliefs or conditions assumed to be present that are accepted as true or assumed to exist for the logic model to operate as intended. These are not necessarily presented in detail but are significant since they may have an impact on the effectiveness of the intervention. Typically, assumptions can be of different aspects such as, for instance, contextual conditions, stakeholders’ involvement, availability of resources, changes in behavior, and casual links etc. (73). Assumption regarding the contextual conditions are such as community, culture, or environment where intervention is going to occur. For instance, in an anti-bullying model, an assumption can be such that there is a common cultural value of tolerance and respect between students and teachers. Assumption regarding the stakeholder involvement include stakeholders’ involvement and commitment, e.g., parents, teachers, and administrators. For example, an assumption could be that teachers will participate in training programs or that parents will collaborate in anti-bullying programs. Assumption regarding availability of resources include availability of resources, e.g., funding, staffing, or materials. For instance, the model can hypothesize that the school can arrange counselors or there will be sufficient budget to allocate for anti-bullying programs and campaigns. Assumption regarding behavior change include how individuals (e.g., students, teachers, parents) will react to the intervention. For instance, one of the assumptions in an anti-bullying logic model could be that if bullying awareness programs are carried out, then students will be aware of the effects of bullying and will be more likely to report. Assumption regarding causal pathways involves cause-and-effect between activities, outputs, and outcomes. For example, the model may presume that training teachers on how to respond to bullying incidents will automatically decrease bullying instances, or that enhancing reporting procedures will lead to more bullying cases being reported. External or contextual/environmental factors are those outside the stakeholders’ control of the program or intervention but may have considerable impact on the results of the logic model (74). They may facilitate or hinder the success of the intervention. For instance, the government policies and political climate may affect the execution of the logic model. For instance, if there is no government support for anti-bullying programs or policies change, this may influence the effectiveness of the intervention. Likewise, if there are laws requiring bullying prevention programs, this may serve to create greater implementation motivation. Second, there are social and cultural forces that can influence the intervention.

The broader cultural or societal attitudes toward bullying, violence, or discipline in schools play a significant role in the intervention’s effectiveness (2). Bullying can be more accepted in some cultures and firmly rejected in others. These societal views can influence how parents, students, and teachers interact with anti-bullying programs. Thirdly, the availability of financial resources tends to be an outside variable influencing the effective enforcement of an intervention (75). Budget may restrict the scale of the anti-bullying programs, for example, the extent of training programs or services available. Financial conditions may impact the funds students and families can access, which may, in turn, impact their capacity to participate in the program (76). Fourthly, if school administrators do not make bullying prevention a priority or do not provide needed resources, the program can be challenging to implement and sustain (77). The technological environment has been found to have a direct impact, i.e., social media sites and cyberbullying, could extend to bullying conduct beyond the classic school environment. Some of these factors may not be met with an optimal fit by a physical-school-focused, conventional anti-bullying model (78). Furthermore, legal and regulatory considerations like laws or rules concerning education, students’ rights, or the definition of bullying can influence how to conduct the intervention (79). For instance, in the event of having specific reporting protocols to be done in the case of bullying incidents, or in case the legal provisions for the protection of students are incomplete, then the model must be revised to suit those specifications. Lastly, community and public opinion also matter because how the community sees bullying and what the school does to deal with it affects the success of the logic model (80). If the community does not think bullying is a big deal, or if there are unwillingness to intervene, the model might have to include other community engagement strategies (81).

In short, assumptions and external factors both contribute to establishing the context in which the logic model is to be applied. While assumptions determine what expectations are made regarding how the intervention will function, external factors offer a wide-ranging context that could impact the success of the intervention. Both must be similarly carefully taken into account in the design and implementation to enhance the chance of the desired outcomes being realized. Challenges of developing and using Logic models are a flexible approach to developing a working theory of what may happen during an intervention. One concern articulated around the use of logic models is “what if the initial theory is wrong?” (82). Kneale et al., further declare that this is a legitimate concern. But stakeholder involvement strategies and using logic models to draw on existing theory, can reduce this risk. Logic models which use more general social and other theories in informing their construction can reduce the risk of developing an incorrect or irrelevant theory (29). In addition, the methods surrounding planned iteration of the logic model to account for evolving knowledge around the intervention (and recording this process) results in theory being investigated, refined, and enhanced as a consequence of the review. Lastly, as argued above, logic models for anti-bullying program are routinely employed to present and explain the results of the synthesis (83), and for certain types of anti-bullying program, logic models will imply, more than “test”, a theory. Logic models can rapidly turn complicated, to the point that they lose clarity and become far too complex to offer a rigorous framework for examination (84). This is a valid criticism, and although the equifinality principle will probably apply to most complex interventions, large numbers of varying combinations of possible pathways become impossible to manage for analytical or communication reasons. Once again, stakeholder engagement can assist in prioritizing pathways in a logic model; in addition, one must think of a logic model as a simplified version of a complex intervention system.

Development of a logic model involves dealing with this complexity in a way that ensures it reflects those routes hypothesized as being the most critical. Stakeholder engagement and public participation when creating a logic model is crucial (85). It is extremely essential to find and engage suitable stakeholders from the beginning while constructing a logic model, especially to make the salience of the model stronger and to develop a sensible and useful model for all stakeholders (86). Stakeholders may provide lived experience of a specific health problem (e.g., patient, carer, parent or relative), might lobby on behalf of those with lived experience (e.g., patient groups), or might offer professional or acquired knowledge (e.g., practitioners, clinicians, researchers and policy-makers). Among their other advantages, stakeholder engagement can test hypotheses regarding the intervention, or the condition being studied (82, 86) guarantee diversity of opinion are included; and assist with the identification of contextual factors that support or impede the implementation of the intervention (29). Since logic models may be visually compelling and interactive, both in the process of creation as well as in the resulting product, they lend themselves to the participation of various stakeholders. In addition, since the role that logic models can play in shaping the review, decision-making throughout the review, and in presenting the findings is so important, engaging stakeholders in the development of logic models may be a way of preventing tokenistic stakeholders’ engagement (29, 86).

Developing logic models can go further to ensure that the logic model is developed in a way that is inclusive and human and that challenges world views (87, 88). Regardless of approach, involving stakeholders in developing logic models should avoid the need for one model that supports decisions and communication within the team conducting the anti-bullying policy-making, for example, and a separate public-facing model aimed at stakeholders—and beyond. A single model serves a dual purpose as a tool and a medium for transparently conveying assumptions and evidence beyond the team. Equity, Diversity and Inclusion Logic models offer potential as tools to support further consideration of equity issues (89). Current logic model practice often “lumps together” equity considerations as potential effect modifiers, but the prospect for a greater role for logic models to support theorizing about these distributional impacts remains. General and intervention-specific equity logic models have been proposed structured around Recruitment, Intervention and Outcome Evaluation for an intervention or program (90). In addition, reflexivity Stakeholder biases could influence the development and evolution of logic models (91). Given the importance that logic models can play in bullying, the role of stakeholder perspectives and potential conflicts of interest needs to be made transparent, as these can influence the direction of interpretation and communication of findings. In addition, transparent reporting of how stakeholders contributed to the development of the logic model, as well as transparent reporting of how the logic model contributed to the direction of the review, could help to understand how such conflicts of interest could influence the findings. Where clear conflicts of interest or divergent perspectives exist among particular groups who contribute to the development of the logic model, there is a case for publishing a separate logic model that clearly shows these distinctions.

Finally, although no direct supporting documents are available for direct comparison between the previous and the present antibullying logic model, but based on existing literature and our research questions, a comprehensive discussion is presented in the following section to address the research questions formulated for this study.

*What are the primary elements of a sound anti-bullying logic model for Abu Dhabi schools?*:

The logic model highlighted critical elements—inputs, activities, outputs, outcomes, and impacts—that are consistent with international anti-bullying models (92) and that respond to the UAE’s distinctive cultural, institutional, and policy context. In contrast with much Western-based models stressing punitive action, the model gives priority to stakeholder engagement, education reform, and social–emotional learning and is consistent with research by Hong and Espelage that puts stress on concerted strategies in bullying prevention in schools (93).

*What do* var*ious stakeholders perceive about current anti-bullying programs and how can they be improved?:* The stakeholders, such as educators, government representatives, and child protection professionals, complained about existing top-down solutions. They called for more participatory approaches that include students and parents in program planning and evaluation—an observation substantiated earlier on (94), which highlighted stakeholder ownership in the declination of bullying episodes. This convergence advised the model’s participatory design and its focus on community endorsement.

*How can participatory methods help develop a locally relevant and sustainable intervention?*: The integration of participatory research methods significantly enriched the logic model. Engaging stakeholders through FGDs and interviews ensured the model reflected real-world constraints and priorities. Other such participatory models have been successful in various contexts, e.g., in the U. S. and South Korea, where collaborative planning resulted in greater program adoption and sustainability (95). The Abu Dhabi model is therefore adding to the mounting evidence that participatory co-design makes school-based interventions more relevant and successful.

*What are the anticipated short- and long-term outcomes of applying this model?*: Short-term results comprise heightened awareness, enhanced reporting, and more cohesive school-level policy coherence. Medium- and long-term results should comprise fewer instances of bullying, healthier mental well-being among students, and improved safety within schools. These correspond with global research highlighting that well-designed, whole-school strategies can diminish bullying by as much as 20–30% in the long term (96).

Compared to current anti-bullying literature, this research is an uncommon illustration of a localized, systems-thinking model being implemented within a Middle Eastern setting. Most international models are based on Western nations and frequently overlook the socio-cultural dynamics that drive bullying behaviors and reporting in other settings (97). By basing the model on local realities and engaging ministries, educators, and parents, this study closes that gap and provides a copycat framework for culturally adaptable interventions.

Even with the detailed design and co-production of this study, a number of limitations need to be noted. First, the logic model was established based on a purposive sampling of stakeholders based mainly in Abu Dhabi, which might restrict generalizability to other emirates or other areas with varying institutional and cultural backgrounds. Second, although the study included a variety of data collection modes—interviews, focus group discussions, and analysis of documents—the project was heavily dependent on qualitative findings, and therefore there is a possibility of subjective interpretation or prejudice. Third, the model was constructed based on a point in time and perhaps does not depict fully changing dynamics in school settings, changes in policies, or alterations in societal attitudes toward bullying. In addition, because of the non-empirical basis of the first phase, the model has not yet been piloted in large-scale rollout or longitudinal analysis, and its longer-term effects have yet to be empirically confirmed. Finally, although attempts were made to engage with diverse stakeholder views, some viewpoints—again, those of students or other traditionally marginalized groups—were potentially under included.

## 5 Conclusion

This participatory approach resulted in a strategic, culturally appropriate anti-bullying logic model. The model offers a roadmap for sustainable implementation and evaluation across schools in the region. The development of this Comprehensive School Anti-Bullying Logic Model in Abu Dhabi represents a thoughtful and culturally tailored approach to addressing bullying in schools. By using a multi-method approach and engaging a wide range of stakeholders, the study has produced a framework that is grounded in both local realities and international best practices. This model provides a strategic guide for schools to implement effective policies and programs to reduce bullying and create a safer, more supportive educational environment. The study underscores the importance of collaborative efforts in addressing complex issues like bullying, ensuring that the solutions are practical, relevant, and sustainable.

## Data Availability

The raw data supporting the conclusions of this article will be made available by the authors, without undue reservation.
