# Correction: Development of a comprehensive school anti-bullying logic model in Abu Dhabi: a multi-method participatory approach

**DOI:** 10.3389/fpubh.2025.1708227

**Published:** 2025-10-13

**Authors:** Alfan Al-ketbi

**Affiliations:** Department of Health Care Management, College of Health Sciences, University of Sharjah, Sharjah, United Arab Emirates

**Keywords:** school-bullying, logic models, mental health, academic performance, UAE

In the published article, there was a mistake in the **Acknowledgments** section. It originally stated: “Thanks to all experts for their participation and guidance”. The correct statement is “The author gratefully acknowledges the valuable support of the team from the Institute of Public Health, College of Medicine and Health Sciences, United Arab Emirates University, including Prof. Michal Grivna and Dr. Iffat Elbarazi. The author also extends sincere appreciation to participating stakeholders and institutions for their openness and valuable insights in addressing bullying issues in the UAE.”

The original article has been updated.

