# Supplementary material for: Development of a comprehensive school anti-bullying logic model in Abu Dhabi: a multi-method participatory approach
Source: Front Public Health. 2025 Aug 20;13:1649884. doi: 10.3389/fpubh.2025.1649884 (PMC12405265; doi:10.3389/fpubh.2025.1649884)
Supplement: Supplementary file 1 [file Table_1.docx]

**Supplementary File 1**

**Positive/Critical Comments used to further refine the model**

| **Feedback Category** | **Positive Comments** | **Critical Comments** |
| --- | --- | --- |
| **Structure & Clarity** | …model is logically sequenced and well-organized | Some overlapping or |
|  |  | ambiguously defined outcomes and activities |
| **Evidence Base** | Global best practice and stakeholder considerations included | …….insufficient transparency in synthesis of local data into model components |
| **Cultural Relevance** | Displays some sensitivity to the UAE context through school policies | not adequately addressed: cultural diversity and contextual behaviors. |
| **Stakeholder Involvement** | Multi-level participation involved (students, teachers, parents). However, there is further work needed on stakeholder identification and may be their network analysis | Minimal evidence for DIRECT student or parent involvement in development. |
| **Implementation Feasibility** | Workshop and training activities are realistic and feasible | Resource and school readiness assumptions are not clearly stated. |
| **Outcomes & Indicators** | tShort- and long-term outcomes with partial measurable indicators. | More explicit link required between resources and long-term outcome (e.g., lowered rate of bullying). |
| **Innovative Features** | Meets prevention, education, and policy in an integrated strategy | Redundancy among pathways among some activities and outputs. |
