# Supplementary material for: Development of a comprehensive school anti-bullying logic model in Abu Dhabi: a multi-method participatory approach
Source: Front Public Health. 2025 Aug 20;13:1649884. doi: 10.3389/fpubh.2025.1649884 (PMC12405265; doi:10.3389/fpubh.2025.1649884)
Supplement: Supplementary file 2 [file Table_2.docx]

**Supplementary Files**

**Table 1:** **Key components of the Logic Model – Inputs/Resources**

| **Inputs/Resources*** | **Importance and Justification** |
| --- | --- |
| Human Resources: Trained staff, including counselors, anti-bullying coordinators, and teachers, who are equipped to implement the interventions. | Human resources constitute a critical input in the implementation of any successful anti-bullying program. The success of the Comprehensive School Anti-Bullying Logic Model relies, in large part, on the capacity, training, and dedication of the personnel working on the interventions' implementation, management, and maintenance. They must possess technical knowledge, but also the interpersonal and emotional capacities to manage difficult issues pertaining to bullying. |
| Educational Materials: Resources for educating students and teachers on identifying and addressing bullying, including training materials, educational programs, and anti-bullying campaigns. | Educational materials are a vital input in ensuring effective application of the Comprehensive School Anti-Bullying Logic Model. They are the materials and content used to instruct students and teachers on bullying, its impact, and how to successfully prevent and manage it. Effective materials provide a common understanding among all members of the school community and give them the knowledge to take preventive action. |
| Financial Support: Budget allocation for the implementation of anti-bullying programs, such as workshops, support services, and awareness campaigns. | Financial assistance is a key input towards the success of any anti-bullying program. Proper funding guarantees that all activities and inputs required by the program can be obtained and sustained throughout the lifespan of the program. Without the right financial support, even quality programs can falter in attaining their intended objectives. Allocation of funds includes important areas such as staff development, production of materials, provision of support services, and campaign awareness, all of which are important towards the creation of a long-term, effective effort towards bullying prevention. |
| Community Engagement: Involvement of parents, local community leaders, and external experts who can provide support and contribute to the intervention’s success. | Community involvement is an essential ingredient for the effectiveness of an anti-bullying program because it creates a participative climate in which everyone involved—teachers, students, parents, leaders in the community, and outside experts—is responsible for the development of a safe and welcoming school environment. Engaging the larger community not only maximizes the effect of anti-bullying efforts but also guarantees their long-term sustainability. When community members are actively involved, it fosters a collective sense of responsibility for preventing bullying and builds a robust support network for students and staff. |

**Table 2:** **Key components of the Logic Model – Activities**

| **Activities** | **Importance and Justification** |
| --- | --- |
| *Human Resources:* Trained staff, including counselors, anti-bullying coordinators, and teachers, who are equipped to implement the interventions. | The effectiveness of the anti-bullying intervention is very much dependent on the presence of qualified and trained personnel, including: • Experienced school counselors who specialize in conflict resolution and student mental health. •School-level designated anti-bullying coordinators who are responsible for the organization of school-wide approaches and as a contact point for teachers, students, and parents. • Trained educators who are sensitized to see, respond to, and avert bullying behaviors through classroom management and positive behavioral interventions. These human resources are the backbone of the program and provide assurance that evidence-based practices are effectively translated into daily school operations. Their professional development is also a major consideration for maintaining program outcomes. |
| *Educational Materials:* Resources for educating students and teachers on identifying and addressing bullying, including training materials, educational programs, and anti-bullying campaigns. | An integrated collection of educational materials is necessary to facilitate awareness, understanding, and action against bullying. Such materials consist of:   - Teacher training materials and odules specifically designed to prepare teachers with methods to detect, avoid, and react to bullying in educational environments. - Student-focused educational programs aimed at developing empathy, communication, and peer support systems. - Print and electronic materials, such as posters, activity booklets, and presentations that encourage positive behavior and anti-bullying messages. - Anti-bullying campaign toolkits, such as slogans, media materials, and event planning guides for school-wide and awareness week initiatives. These tools guarantee messaging consistency, support a culture of respect, and empower teachers and students to become active contributors to a safe and inclusive school climate. |
| Financial Support: Budget allocation for the implementation of anti-bullying programs, such as workshops, support services, and awareness campaigns. | Sustainable and effective rollout of anti-bullying programs needs committed funding to promote continuity and accessibility. Funding encompasses:   - Educational authorities or sponsor organizations' allocated budgets (e.g., government, NGOs, or UNICEF) to cover intervention programs. - Funding workshops and training sessions for capacity development among students, counselors, and school staff. - Student support service funds, including counseling, peer mentoring schemes, and helplines. - Awareness campaign costs, which cover materials, events, and community activities. - Strategic money investment guarantees scalability, quality, and long-term effectiveness of anti-bullying interventions in schools in the region. |
| Community Engagement: Involvement of parents, local community leaders, and external experts who can provide support and contribute to the intervention’s success. | The effectiveness and sustainability of anti-bullying programs are significantly increased through active community engagement, which establishes a supportive environment around the school. The main elements are:   - Parental engagement through frequent communication, awareness programs, and parenting workshops to support positive behavior at home. - Involvement of local community leaders and influencers who can promote respectful, non-violent behavior and support anti-bullying initiatives in the wider community. - Involving external experts such as psychologists, child rights champions, and social workers who are able to give technical assistance, training, and crisis intervention on a need basis. - Creating school-community partnerships through which to co-design initiatives which are informed by local values, cultural sensitivities, and co-responsibility for children's well-being.   This partnership assists in the infusion of anti-bullying values not only within school culture but also in the broader community setting, building shared ownership and support. |
| Teacher Training: Regular workshops to equip teachers with the skills to recognize, respond to, and prevent bullying. These workshops aim to create a culture of inclusivity and respect within the classroom. | In order to create an active and responsive school culture, the intervention provides ongoing, organized workshops for teachers that address:   - Identifying indicators of bullying, including insidious types like social exclusion and cyberbullying. - Successful intervention tactics, enabling teachers to act assertively on incidents in a timely and supportive fashion. - Preventive classroom management strategies, including encouraging empathy, inclusivity, and respectful communication among students. - Building a safe classroom environment, in which all students are valued, supported, and heard.   These workshops not only build teacher capacity but also support school culture based on respect, equity, and student well-being. |
| Student Awareness Campaigns: Programs designed to raise awareness about the different forms of bullying (physical, verbal, social, cyberbullying) and the impact it has on individuals and the community. | One of the main pillars of the intervention is the enforcement of extensive awareness campaigns among students, aimed at developing understanding, empathy, and responsibility. These campaigns emphasize:   - Raising awareness among students regarding the different types of bullying—physical, verbal, social, and cyberbullying—and how to identify them. - Emphasizing the emotional, psychological, and academic effects of bullying on victims, peers, and the wider school community. - Fostering positive behavior modification through peer-organized activities, school assemblies, creative arts (posters, drama, debate), and participatory sessions. - Student-led advocacy, where students are actively involved in developing anti-bullying messages and sustaining a safe school culture.   The campaigns aim at empowering students to be well-informed, responsible, and empathetic, and thus creating a school culture in which bullying is actively discouraged and addressed in a timely manner. |
| Peer Support Programs: Establishing peer mentoring and support networks to help students feel more empowered to report bullying and support their peers. | These initiatives seek to equip students as change agents in the prevention and resolution of bullying through the establishment of formal peer support systems. Key elements are:   - Peer mentoring programs where student mentors who have received training offer guidance, emotional support, and an ear to listen to other students. - Buddy systems to facilitate the integration of new or vulnerable students and prevent social isolation. - Student support groups or clubs led by students to foster inclusion, raise awareness, and promote peer advocacy. - Training sessions for peer supporters, with the aim of empowering them with conflict resolution skills at a basic level, active listening, and referral skills.   These initiatives help build a sense of belonging, empathy, and responsibility, making the students active agents in promoting a safe and respectful school climate. |
| Clear Reporting Mechanisms: Creating and promoting safe, anonymous channels for students to report bullying incidents without fear of retaliation. | To guarantee that incidents of bullying are taken care of in a timely and effective manner, intervention entails building and promoting available and confidential reporting mechanisms. This entails:   - Creating a variety of reporting means, including face-to-face reporting to confidant staff members, anonymous drop-boxes, and online platforms or hotlines. - Assigning trained staff (e.g., counselors or anti-bullying coordinators) who will take and process reports with care and discretion - Organizing awareness sessions to enlighten students regarding their right to report bullying and how to do so. - Providing safeguards to prevent retaliation and ensure student safety and confidentiality.   These mechanisms enable students to report bullying and seek assistance, supporting a school culture of not tolerating bullying and respecting student well-being. |
| Parent Involvement: Engaging parents in the anti-bullying efforts through informational sessions and collaboration on strategies to support their children at home. | In acknowledging the role of families in influencing children's attitudes and behaviors, the intervention has active parent involvement through:   - Information workshops and sessions to inform parents about the occurrence of bullying as victim and perpetrator, and how to assist their children effectively. - Dispensing educational materials like brochures, newsletters, and online content that reinforce anti-bullying messages and techniques. - Ongoing communication channels between homes and schools to share progress, discuss concerns, and establish trust. - Parental involvement in school events, campaigns, and committees concerning student welfare and anti-bullying efforts.   This combined effort guarantees congruence of school and home environments, supports early identification of problems, and enables parents to become active collaborators in creating secure and supportive learning environments. |

**Table 3: Key components of the Logic Model - Outputs**

| **Outputs** | **Importance and Justification** |
| --- | --- |
| The number of teachers trained in anti-bullying practices. | Specific information on the precise number of teachers who have been trained in anti-bullying techniques within the United Arab Emirates (UAE) is not immediately available. Nevertheless, the government of the UAE has shown significant intent to combat bullying among schoolchildren through numerous programs:   - National Bullying Prevention Strategy: This was created in 2018 and entails various ministries conducting bullying prevention interventions nationwide. Injury Prevention (<https://www.moe.gov.ae/En/MediaCenter/News/Pages/Bullying-Prevention.aspx>) - National Bullying Prevention Week: Initiated in April 2018, this campaign involves workshops, lectures, and awareness drives at public and private institutions. These sessions are conducted by experts and consultants who train students and teachers on how to identify and tackle bullying. Ministry of Education UAE+1Ministry of Education UAE+1 (<https://www.moe.gov.ae/En/MediaCenter/News/Pages/Bullying-Prevention.aspx>) - Creation of Educational Materials: The Supreme Council for Motherhood and Childhood in partnership with UNICEF established a detailed national intervention program addressing the UAE setting. This has a 'Manual for - Bullying Prevention' accompanied by intervention approaches and social skills development activities. BPA+1Ministry of Education UAE+1 (<https://u.ae/-/media/Information-and-services/Justice-safety-and-the-law/Guide-for-parents-on-anti-bullying.pdf>)   These initiatives imply that many educators have been trained in anti-bullying. Yet, short of having figures, it is difficult to quote an exact number. |
| The establishment of reporting channels for bullying incidents. | In the UAE, the creation of reporting mechanisms for bullying cases has been a central theme of anti-bullying initiatives. Several initiatives have been launched to facilitate reporting by students, parents, and school personnel and to access support. Some of the most important initiatives are:   - Ministry of Education (MoE) Reporting Channels: The MoE has established a range of reporting mechanisms for bullying in schools. This consists of special hotlines, online reporting platforms, and email addresses through which parents, students, and teachers can report bullying behavior anonymously. The MoE also recommends that schools create their own internal reporting mechanisms so that they can respond quickly. - Student Protection Hotline: The UAE has also set up a Student Protection Hotline as part of its child protection policy. This enables students, parents, and teachers to report bullying and other abuse directly to the concerned authorities. The hotline is available 24/7 and provides a confidential way of reporting bullying issues throughout the country. - School-Based Reporting Mechanisms: Most schools in the UAE have established their own reporting mechanisms. These may be in the form of online complaints, specific staff members (like school counselors), or school management personnel who deal with bullying complaints. Students are taught to report bullying to trusted adults in the school setting. - Anti-Bullying Policies: The UAE has implemented anti-bullying policies, which contain specific guidelines for reporting bullying. These policies mandate that schools establish formal reporting and intervention systems as well as follow-up measures to address problems. - Dubai Police's "No to Bullying" Campaign: Dubai Police has initiated awareness campaigns in an effort to raise awareness about bullying and get people to report it. Part of these campaigns includes various reporting mechanisms, such as mobile applications and specialized online platforms, where bullying incidents can be reported anonymously.   These initiatives are designed to ensure that bullying incidents are reported and addressed in a timely and effective manner, fostering a safer and more supportive environment for students across the UAE (<https://u.ae/-/media/Information-and-services/Justice-safety-and-the-law/Guide-for-parents-on-anti-bullying.pdf>)  . |
| The number of students who participate in bullying awareness programs. | Although individual participation figures in bullying awareness programs in all UAE schools are not easily available, a number of initiatives have been undertaken to increase awareness and deal with bullying among students:   - National Bullying Prevention Week: Conducted by the Ministry of Education in partnership with the Supreme Council for Motherhood and Childhood, the event is conducted annually and includes workshops, lectures, and awareness campaigns in public and private schools. The second campaign from November 18 to 24, 2018, saw trained teachers and consultants conduct visits to schools to inform students, teachers, and parents of bullying prevention. Ministry of Education UA - Bullying Prevention Program: Launched by the Supreme Council for Motherhood and Childhood, the program was rolled out to 61 public and private schools in the 2015-2016 academic year. Students, school principals, academic supervisors, and nurses were the targets of the program, seeking to enhance capacity building and raise awareness regarding bullying. scmc.gov.ae   These efforts emphasize the UAE's dedication to providing a secure and nurturing learning environment by proactively involving students, teachers, and parents in anti-bullying awareness and prevention activities. (<https://u.ae/-/media/Information-and-services/Justice-safety-and-the-law/Guide-for-parents-on-anti-bullying.pdf>) |
| The number of parents attending anti-bullying workshops. | Where precise attendance statistics about anti-bullying workshops among parents in the UAE are not available, a few initiatives have sought to engage parents actively in their anti-bullying efforts:   - National Bullying Prevention Week: Conducted by the Ministry of Education, this takes the form of workshops and awareness sessions with the participation of students, parents, principals, and educators. At one session, 57 people worked together and prepared recommendations to fight bullying. Ministry of Education UA - Parent Guides and Resources: Parent guides from the UAE government enable parents to identify and handle bullying. They include tips for dealing with the impacted children as well as learning legal implications. UAE.gov.ae - Parental Involvement Programs: The Supreme Council for Motherhood and Childhood has collaborated with UNICEF and established programs engaging parents in response to bullying. The programs intend to strengthen relationships between schools and families and enable parents to get trained in identification and response towards bullying. ResearchGate+2PubMed+2BioMed Central+2   These efforts emphasize the UAE's interest in involving parents in making a school environment safer by actively including them in anti-bullying prevention and intervention efforts. (<https://u.ae/-/media/Information-and-services/Justice-safety-and-the-law/Guide-for-parents-on-anti-bullying.pdf>) |
| Creation of support groups for both victims and perpetrators of bullying, providing ongoing support. | In the UAE, establishing support groups for victims and bullies alike is one of the comprehensive ways to ensure that every student gets the guidance and care they need. The measures seek to address bullying not only on the level of prevention but also on intervention and continuous support to all stakeholders. (<https://u.ae/-/media/Information-and-services/Justice-safety-and-the-law/Guide-for-parents-on-anti-bullying.pdf>)  Key Initiatives and Programs:   1. Counseling and Psychological Support:  - Numerous schools in the UAE have incorporated counseling services to address the emotional well-being of bullying victims and perpetrators. Students are assisted by school counselors to comprehend their behavior and emotional reactions, providing individualized support to enhance social skills and emotional control. - Special programs can also be provided to students who engage in bullying for them to acknowledge the implications of their actions and to promote positive behavioral modification.  1. Peer Support Programs:  - Other schools have also introduced peer support groups, with trained students as mediators or assistants to bullying victims. The peer-led projects assist in promoting a culture of respect and understanding among peers as well as improving empathy and conflict resolution skills. - The peer support groups also provide bullies with an avenue for realizing that their behavior is not threatening, as they can learn from their peers on how to interact better with other people.  1. Parent and Teacher Support:  - Regular workshops and training for parents and teachers are organized, focusing on creating supportive environments for both victims and perpetrators of bullying. These sessions help caregivers and educators understand how to handle such issues and provide consistent emotional and social support to the children involved. - This collaborative approach ensures that the children receive guidance both at school and at home.  1. Online Support Platforms:  - Efforts have been made by the UAE to provide anonymous reporting portals for bullying attacks. Such platforms not only enable victims to report bullying with impunity but also assist in linking families to service provision. - Certain portals might offer resources for victims as well as bullies, such as psychological counseling, conflict resolution techniques, and manuals on enhancing social conduct.  1. National Anti-Bullying Programs:  - Initiatives such as the National Bullying Prevention Week may incorporate activities wherein victims and those who display bullying behavior are helped through group therapy and workshops that both heal and correct behavior. - Perpetrator-specific programs might involve formalized behavioral therapy, and victims could be provided with therapy and support groups to overcome trauma.  1. Institutional and Legal Support:  - In extreme cases, there are avenues for victims and their families to pursue through law. The UAE has attempted to assist both victims and perpetrators through educational reforms and legislation designed to prevent bullying and provide continued psychological support. - By addressing bullying from both sides—helping victims to heal and helping perpetrators to modify destructive behavior—the UAE is hoping to create a more compassionate and safe learning environment for all students. |

Outputs are the immediate, tangible results of these activities.

**Table 4: Key components of the Logic Model - Outcomes**

| **Outcomes** | **Importance and Justification** |
| --- | --- |
| Improved Awareness: Increased awareness among students, teachers, and parents about the prevalence, effects, and prevention of bullying. | Increased awareness regarding the prevalence, impact, and prevention of bullying has been a major area of concentration of anti-bullying initiatives in the UAE to provide a safe and friendly environment for students, teachers, and parents.  Raising awareness and involving all stakeholders in the fight against  bullying has been addressed by a number of initiatives. Major Awareness-Building Initiatives:   1. National Bullying Prevention Week:  - Purpose: This program, held each year by the Ministry of Education (MoE), unites students, instructors, and parents to increase bullying awareness. It involves several activities like workshops, discussions, and awareness campaigns. - Effect: These efforts have served to educate participants concerning the impact of bullying and why prevention measures are crucial, reaching out to a wider school population in fighting against bullying.  1. Teacher Workshops and Training:  - Focus: Teachers are taught to identify signs of bullying, its effects, and effective strategies for preventing and responding to it. - Scope: These workshops usually involve role-playing, case studies, and discussions to enable teachers to enhance their response to bullying. Teachers are also taught how to create an inclusive and supportive classroom environment. - Outcome: Through improved understanding by teachers, such workshops raise the capacity of schools to deal effectively and immediately with incidents of bullying.  1. Parent Education Programs:  - Purpose: Parents receive materials and education on how to recognize bullying behaviors in their child and how to respond effectively if their child is being bullied, as a victim, or doing the bullying, as an offender. - Tools: This usually comprises details regarding the emotional, psychological, and social impact of bullying, and guiding parents on how to approach discussing bullying prevention with their children. - Effect: Parents are made to be proactive in facilitating prevention of bullying, as well as in being supportive to their children during difficult times.  1. Peer-Led Awareness Programs:  - Student Role: Peer support programs make students active participants in raising awareness among their peers about bullying. Trained student leaders or peer counselors can organize campaigns, design posters, or engage in group discussions to spread anti-bullying messages. - Advantages: These programs make students feel more committed to the cause and promote a culture of inclusiveness and respect for one another.  1. National Media Campaigns:  - Campaigns: Schools and the UAE government have used media outlets, such as TV, social media, and websites, to promote anti-bullying messages. The campaigns emphasize respect, kindness, and inclusiveness and inform the public about the harmful impacts of bullying. - Wider Reach: Media campaigns aid in raising awareness among a greater population, such as the public and community members, who may not be immediately engaged in schools.School  1. Policies and Initiatives:  - Anti-Bullying Policies: Numerous schools in the UAE have implemented anti-bullying policies that involve specific guidelines on how to identify, report, and prevent bullying. Such policies tend to focus on raising awareness through curriculum integration and school events. - Curriculum Inclusion of Bullying: Some schools integrate bullying, empathy, and social emotional learning into the curriculum. This educates students about why they need to treat others with respect from a young age  1. Support and Awareness Platforms:  - Hotlines and Online Resources: The UAE has established online portals, hotlines, and applications where students, teachers, and parents can access bullying information, report cases, and receive guidance. These portals facilitate more awareness and a confidential environment for individuals to receive help. - Anonymous Reporting: These websites enable students to report incidents of bullying without being afraid of repercussions, allowing the issues to be addressed quickly.  1. Impact of Increased Awareness:  - Understanding and Empathy: Empowerment programs generate empathy among children and teachers so that it is easier to spot the signs of bullying and stop it early on. - Prevention: Greater awareness aids in stopping bullying by teaching students and instructors the importance of being inclusive and respectful. It also teaches parents how to identify children who could be victims of bullying. - Culture Change: The aim is to develop a school culture in which bullying is not acceptable and in which teachers, students, and parents collaborate to ensure a safe and healthy learning climate.   Through increased awareness of bullying and its impact, these efforts are crucial in bringing about long-term change in attitude and behavior towards bullying in the UAE. |
| Higher Reporting Rates: A higher number of bullying incidents being reported by students due to improved reporting mechanisms and a stronger culture of openness and support. | In the UAE, improvement in reporting mechanisms and creating a culture of openness and support has resulted in increased rates of reporting of bullying episodes. Such initiatives are aimed at making students feel secure and sure about reporting bullying and ensuring that schools are able to respond in a suitable manner. Some of the prominent initiatives responsible for increased reporting of bullying are mentioned below:   1. Anonymous Reporting Systems:  - Purpose: Anonymous reporting systems, including hotlines, mobile applications, or online platforms, have been used by numerous schools and educational institutions in the UAE to enable students to report instances of bullying anonymously. - Impact: Anonymity allows more students to report, since they know their identity will be kept confidential. This has been a factor in increased reporting levels, as students feel safer to report bullying without having to expose their identity.  1. Clear and Accessible Reporting Channels  - School-Based Reporting: Schools have developed clear and accessible reporting mechanisms for bullying, including specific staff personnel (e.g., counselors or anti-bullying officers) who manage these incidents. Most schools also have designated forms or procedures to help guide students to report incidents of bullying. - Impact: The accessibility and simplicity of the reporting process have made more students report bullying when they witness or are victimized by it, realizing that their issues will be addressed and taken seriously in time.  1. Training and Awareness for Students:  - Empowerment: Awareness programs, workshops, and campaigns educate students on the significance of reporting bullying. Students learn how to identify bullying, the effect it has on others, and the significance of reporting it. - Support: Schools foster an atmosphere of support where students are inspired to report instances of bullying and educate them about the fact that reporting the same is a responsible and courageous thing to do. - Outcome: As students are empowered with information regarding reporting bullying and why it is essential, more cases are being reported, and thus the overall reporting rate is higher.  1. Parent and Teacher Involvement:  - Role of Parents: Parents are educated on how to identify signs of bullying and how to report it if their child is a bully. Some schools provide workshops or information sessions for parents to help them assist their children through the reporting process. - Teacher Awareness: Teachers are taught to identify bullying and are also motivated to take an active role in supporting students who report incidents of bullying. This involves providing reassurance and checking up with students to confirm that the incident is dealt with. - Effect: More knowledgeable parents and teachers who understand bullying and the reporting procedure can serve as intermediaries, motivating students to report bullying and ensuring it is dealt with effectively.  1. School Policies and Anti-Bullying Culture  - Anti-Bullying Policies: Most schools have established formal anti-bullying policies that specify the procedures for reporting incidents. Such policies highlight the need to establish a safe environment where students feel free to report bullying without worrying about retaliation. - Supportive School Environment: Schools work to create a culture where bullying is not tolerated, and students feel supported in reporting incidents. Positive reinforcement is given to those who report bullying, and students are reassured that their actions will lead to positive changes. - Impact: This culture of openness and support leads to increased reporting, as students feel that their reports will be handled professionally and with care.  1. Government and Community Support:  - National Initiatives: National programs and awareness campaigns to decrease bullying in schools have been initiated by the UAE government, such as anti-bullying hotlines and websites for reporting incidents by students and parents. - Local Support Networks: Local communities, such as agencies like the Supreme Council for Motherhood and Childhood and child protection agencies, offer assistance and resources for schools in order to tackle bullying. - Effect: With the support of the government and open communication channels, parents and students are more inclined to report instances of bullying since they are aware that measures can be taken against the perpetrators.  1. Follow-Up and Feedback Mechanisms:  - Tracking Cases: Schools are encouraged to follow up on reported bullying cases so they are properly handled. This involves giving feedback to the individual who reported the incident so they can know the follow-through. - Reassurance: By informing and engaging students in the process, schools are able to make students sure their concerns will result in action. - Result: The follow-up procedure reassures students and parents and encourages them to report bullying in the future. Outcome   Due to these enhanced reporting systems and the establishment of a more robust culture of transparency, there has been an evident rise in the reporting of bullying cases in UAE schools. Students are more inclined to report because they believe their concerns will be seriously considered and that there are repercussions for bullying. This heightened reporting enables schools to better deal with bullying, creating a safer learning environment for all students. |
| Behavioral Change in Students: An observable decrease in bullying behaviors as students internalize the messages from awareness campaigns and peer support programs. | 1. In the UAE, changing student behavior towards bullying has been a central objective of the different awareness campaigns and peer support programs in schools. These programs not only seek to increase awareness about bullying but also to bring about lasting attitude and behavioral shifts among students. Observable reduction in bullying behaviors can be traced to the internalization of respect, empathy, and inclusion messages. Some of the most important factors driving this behavior change are as follows: 2. Effect of Awareness Campaigns:  - Understanding Bullying: Awareness campaigns in the UAE have made students aware of the different types of bullying—physical, verbal, and cyberbullying—and how it hurts the person and the community. - Empathy and Respect: Highlighting empathy and respect, these campaigns make students think about the effects of their actions and how important it is to treat others with kindness. The emphasis is on developing emotional intelligence and making students aware of the emotional effect of bullying on others - Outcome: As the students absorb these messages, there is a visible decrease in bullying behavior as they start to interact more respectfully with their peers.  1. Peer Support Programs:  - Peer Role Models: Schools use peer support programs to prompt students to engage in leadership capacities for encouraging favorable behavior. Peer counselors or student leaders who are trained play significant roles in preventing bullying by having their peers appreciate the effects of bullying and educating them on alternative positive behaviors. - Peer Influence: If students witness their peers spreading inclusivity and kindness, the likelihood is greater that they themselves will take similar actions. Peers' positive influence significantly influences behavior, especially within a school setting where norms are most shaped by peer cliques. - Outcome: Such programs have been proven to have a ripple effect, such that the students who would otherwise be engaging in bullying activities are more likely to alter their behavior as a result of the peer-initiated programs.  1. Integration of Social-Emotional Learning (SEL) in the Curriculum  - Focus on Emotional Skills: Social-emotional learning (SEL) is included in the curricula by several schools in the UAE. SEL education helps students learn skills like self-awareness, self-regulation, empathy, and interpersonal skills, all of which are crucial in mitigating bullying behavior. - Conflict Resolution: SEL programs additionally address conflict resolution and instruct students in how to resolve disagreements in healthy and constructive manners. Learning these skills, students are more able to manage situations that could otherwise develop into bullying. - Impact: As students make SEL practice, they are more likely to demonstrate conduct reflecting kindness, patience, and empathy, resulting in a decline in incidents of bullying.  1. Behavioral Interventions for Perpetrators:  - Targeted Support for Bully/Victims: Some schools give targeted support to pupils who bully others through programs that assist them in recognizing the implications of their actions and reform their behavior. These programs usually include counseling, behavior therapy, and providing time for students to review their actions. - Positive Behavior Focus: Instead of emphasizing punishment, these programs reward students for positive behavior by recognizing changes in conduct and offering constructive criticism. - Outcome: Students who have once displayed bullying behaviors can significantly improve as they learn about their behavior and develop healthier means of engaging with others.  1. Teacher and Staff Involvement  - Modeling Positive Behavior: Teachers and school personnel also have a significant role in modeling positive behaviors to students. By being respectful, open-minded, and fair, teachers reinforce the values imparted through awareness campaigns and peer programs. - Active Monitoring and Intervention: Teachers are also taught to monitor student behavior and intervene in incidents of bullying. Through active supervision and intervention, teachers prevent bullying from escalating. - Impact: The active and visible presence of teachers encouraging good behavior establishes the tone for the entire school and helps create a culture of respect and kindness that permeates students' attitudes and behaviors.  1. Parental Involvement:  - Special Home Environment: Schools make a conscious effort to engage parents in preventing bullying by having them attend workshops and discuss the importance of respect and empathy with their children. Involvement by parents reinforces messages students get in school and creates a uniform style of managing bullying. - Outcome: When children notice that both their home and school environments value positive behavior and kindness, they are more likely to adopt these values and exhibit less bullying behavior.  1. Positive School Climate:  - Safe and Supportive Environment: Schools that focus on the development of a positive and inclusive school climate have experienced declines in bullying behavior. These schools foster an atmosphere of acceptance, where differences are valued and students feel safe and supported. - Belonging Focus: Students who sense a sense of belonging and community are less likely to bully. Schools that concentrate on developing close, supportive relationships between students eliminate the social tensions that frequently produce bullying. - Outcome: As the overall school culture becomes more supportive and positive, students are more likely to exhibit behaviors that foster a respectful, non-bullying culture. Observable Reduction in Bullying Behaviors - Decreased Incidents: UAE schools have seen a reduction in bullying incidents due to these extensive efforts. The combined effect of awareness campaigns, peer support programs, SEL education, and positive school climates has resulted in decreased bullying behaviors. - Increased Peer Support: Students are more likely to stand up and support their peers if they see bullying, as the culture of respect and empathy becomes more prevalent. - Long-Term Change: The changes in behavior that are inculcated through these programs are not only fleeting; they seek to bring about long-term change in the way students treat each other, curbing bullying in the long term.   These interventions have produced good results for both lowered rates of bullying and more positive student conduct for the UAE. Through embedding messages in campaigns of awareness and peer support schemes, students add to the overall security, unity of a school community. |
| Stronger School Community: A more positive, supportive school culture where students, teachers, and parents collaborate to reduce bullying and promote inclusivity. | In the UAE, a more cohesive school community has been a primary result of robust anti-bullying programs. Through active engagement of students, teachers, and parents, schools have created a culture of respect, cooperation, and inclusion—setting the stage for sustainable change. This more robust school community is central to preventing bullying and creating an environment where all students feel safe and valued. Key Features of a Stronger School Community in the UAE:   1. Collaborative Anti-Bullying Framework • Whole-School Approach: Schools adopt policies and programs that engage all stakeholders—students, teachers, administrators, and parents—in creating and implementing anti-bullying plans. • Joint Responsibility: Preventing and resolving bullying is not the responsibility of one party; rather, it's a collective responsibility of the whole school community. • Effect: This creates a collective front where everyone is working towards the same objective—safety, respect, and inclusion. 2. Active Parental Involvement • Workshops and Seminars: Parents make regular visits to school-conducted workshops to become aware of signs of bullying, intervention methods, and how they can assist their children. • Parent Councils: Parents are frequently engaged in school decision-making matters concerning student welfare and discipline rules. • Impact: An informed and active parent group enforces anti-bullying values within their homes, maintaining consistency between family and school life. 3. Empowered and Trained Teachers • Ongoing Professional Development: Educators are trained to identify, prevent, and handle bullying, and to promote emotional intelligence and classroom inclusiveness. • Safe Adults: Students recognize teachers as secure, caring adults whom they can go to for help when being bullied or in peer conflict. • Outcome: Educators actively help create trust and a sense of belonging in the classroom, minimizing bullying threats. 4. Student Leadership and Involvement • Peer Support Groups: Students are educated as peer mentors or ambassadors, fostering kindness, empathy, and inclusivity. • Student-Led Campaigns: Schools usually implement student-led campaigns during National Bullying Prevention Week and throughout the year. • Outcome: Students become owners of the school climate and active agents of positive change, which results in fewer bullying cases. 5. Inclusive School Activities and Events • Diversity Days and Cultural Events: Celebrations promoting diversity and respect foster interaction among various student populations. • Cooperation-Based Programs: Cooperation-based and group achievement programs foster unity among students from diverse backgrounds. • Effect: Such activities create a feeling of belongingness, bridging the social barriers that usually create bullying. 6. Open Channels of Communication • Anonymous Reporting Tools: Suggestion boxes and online tools enable reporting without fear, building trust. • Open-Door Policies: Principals promote open communication, where parents and students feel comfortable expressing concerns. • Effect: Transparency and responsiveness enhance the relationships among the school community members and create a supportive atmosphere. 7. Appreciation of Positive Behavior • Positive Reinforcement Programs: Schools have reward programs for kind, respectful, and inclusive behavior. • Celebrating Role Models: Rewarding students who resist bullying or help others reinforce and encourage positive behavior. • Outcome: Encouraging good behavior strengthens peer norms against bullying and reinforces a caring environment. |

**Table 5: Key Components of the Logic Model - Impact**

| **Impact** | **Importance and Justification** |
| --- | --- |
| Reduced Incidents of Bullying: A significant decrease in the frequency and severity of bullying cases within schools. | Perhaps the most concrete evidence of the success of anti-bullying initiatives in the UAE has been a considerable decline in instances of bullying and, particularly, the intensity of these cases. This is a testament to the efficiency of national policies, school-specific programs, and community participation in ensuring safer and more welcoming schools.  Evidence of Decreased Bullying Instances   1. School Reports and Surveys • Numerous UAE schools are now implementing annual climate surveys whereby students, educators, and parents report incidents and experiences concerning bullying. • Data from recent research in schools participating in the National Bullying Prevention Week and campaigns of the same nature have revealed: o Declines in cases of reported bullying year on year. o Lower rates of repetitive or chronic bullying, which signals a reduction in severity. 2. Ministry of Education Monitoring • The UAE Ministry of Education, together with the Supreme Council for Motherhood and Childhood and other organizations, monitors bullying statistics at the national level.( <https://u.ae/-/media/Information-and-services/Justice-safety-and-the-law/Guide-for-parents-on-anti-bullying.pdf>)   • Reports must be submitted by schools, and trends indicate a consistent downward trajectory in physical, verbal, and cyberbullying incidents since the introduction of national anti-bullying initiatives in 2018. |
| Improved Mental Health and Well-Being: Students feeling safer and more supported, which leads to better mental health outcomes, higher academic performance, and increased school attendance. | As anti-bullying campaigns intensify throughout the UAE, a clear and positive influence on student well-being and mental health has been realized. Ensuring safer and more nurturing school cultures has resulted in increased emotional strength, lower levels of stress, and improved academic participation among students. This result speaks to the more profound value of addressing bullying—not only the lack of harm, but the presence of flourishing. Better mental health and well-being is perhaps the most significant and sustained effect of anti-bullying initiatives in the UAE. Through creating safe, inclusive, and emotionally intelligent schools, students are not just encouraged to learn more but to live more. Such transformations resonate beyond the school fence, building a generation that is self-assured, empathetic, and emotionally resilient. |
| Cultural Shift: A long-term cultural change where bullying is no longer tolerated, and respectful, inclusive behavior is the norm across schools in Abu Dhabi. | Perhaps the most revolutionary effect of anti-bullying campaigns in Abu Dhabi is the realization of a cultural change deep-seated within school societies. This transcends cutting incidents down—it's about shifting perceptions, values, and social norms so bullying isn't only sanctioned but essentially untenable in school culture. The cultural change occurring in Abu Dhabi schools is the deepest signal of success for anti-bullying campaigns. It is the transition from intervention to prevention, from policy to principle, and from reaction to transformation. Over time, this culture will not only maintain safe learning spaces but also equip students to lead with compassion in the broader world. |
| National and Global Alignment: The model contributes to the UAE’s broader goals of ensuring the safety and well-being of all children in the educational system, aligning with international standards and child protection laws. | One of the major strengths of the Abu Dhabi Comprehensive School Anti-Bullying Logic Model is its alignment with national imperatives and global frameworks. Incorporating the principles of child protection, well-being, and inclusive education, the model strengthens the UAE's overall vision of a safe, loving, and globally respected education system. Alignment with National Goals and UAE Vision.   1. UAE Centennial 2071 and Vision 2031 • The anti-bullying model directly supports national strategies aimed at: o Enhancing student well-being o Building cohesive and tolerant communities o Providing world-class education in a safe environment 2. Child Protection Framework • The UAE’s Wadeema Law (Federal Law No. 3 of 2016) on children's rights emphasizes the protection of children from all forms of violence, abuse, and neglect. • Abu Dhabi’s model ensures: o Clear reporting mechanisms o Trained staff who understand child protection obligations o Confidential handling of sensitive cases 3. Ministry of Education and ADEK Initiatives • The model supports current efforts by: no The Ministry of Education's Student Well-Being Framework no ADEK's (Abu Dhabi Department of Education and Knowledge) child safeguarding policy • It provides a localized, systematic way to achieve these national standards consistently across schools.   **Alignment with Global Standards and Conventions**   1. United Nations Convention on the Rights of the Child (UNCRC) • The model supports important rights under the UNCRC, including:  - Right to protection from violence (Article 19) Right to education in a safe environment (Articles 28 & 29) Right to be heard in issues concerning them (Article 12)   • This embodies a child-centered philosophy in design as well as in implementation.   1. UN Sustainable Development Goals (SDGs) • Explicitly supports:  - Goal 3: Good Health and Well-Being - Goal 4: Quality Education - Goal 16: Peace, Justice, and Strong Institutions   • By reducing bullying and strengthening inclusion, the model helps fulfill SDG targets for equitable, safe, and supportive learning environments.   1. UNESCO’s Whole School Approach to Preventing School Violence • Abu Dhabi’s model incorporates the same multi-layered elements: o Policy development o School culture change o Stakeholder engagement o Monitoring and evaluation • This ensures global best practices are adapted to local contexts.   Educational and Policy Impact • Standardization of Safe Practices: Schools across Abu Dhabi implement a uniform, evidence-based approach that meets both UAE and global quality benchmarks. • International Credibility: The alignment enhances Abu Dhabi’s reputation as a leader in progressive education reform, especially in student safety and well-being. • Readiness for Global Accreditation: Schools applying for international accreditations (e.g., CIS, IB) benefit from already adhering to child protection and inclusion standards.  Strategic Outcomes  How the Model Dimension Aligns   \| National Child Protection \| Fulfills legal obligations under the Wadeema Law \| \| --- \| --- \| \| Education Reform Goals \| Advances UAE’s aim for safe, high-quality education by 2031 \| \| Global Conventions \| Embeds principles of UNCRC and the SDGs \| \| Best Practices \| Reflects UNESCO, UNICEF, and WHO recommendations for school mental health and safety \| \| Institutional Credibility \| Supports schools in achieving international recognition and compliance \|   The Comprehensive Anti-Bullying Model in Abu Dhabi is not an isolated initiative—it is deeply embedded within national policy frameworks and globally recognized child rights standards. This alignment ensures the model is sustainable, scalable, and impactful, contributing meaningfully to the UAE’s long-term vision of providing a safe, inclusive, and globally competitive educational experience for every child. |

The Logic Model also gives assumptions and external factors as shown in Table 2 and Table 3, respectively with relevant details.

**Table 6: Assumptions of the Logic Model**

| **Assumptions:** | **Importance** | **Risk if the Assumption Fails** |
| --- | --- | --- |
| Teachers and students will engage in and commit to the anti-bullying initiatives. | This assumption is integral to the success of the Comprehensive School Anti-Bullying Logic Model. The success of awareness campaigns, peer support systems, reporting systems, and school-wide policies is largely dependent on the active engagement and commitment of both students and teachers. | If this assumption does not prove to be correct, the model might struggle with: •Resistance from teachers who perceive themselves as being overworked or inadequately trained. • Lack of student enthusiasm or conviction regarding the value of anti-bullying efforts. • Token participation, where programs are implemented but not internalized. Therefore, an effort needs to be made to: • Train and motivate teachers properly. • Empower students to envision themselves as leaders and guardians of school culture. • Maintain continuous communication, support, and acknowledgment for those involved. |
| There will be sufficient resources (financial, human, and material) to carry out the activities. | This assumption responds to operational feasibility in instituting the Comprehensive School Anti-Bullying Logic Model. Adequate resources need to be in place to make interventions that are scheduled possible and sustainable. | Without adequate budgeting, activities may be delayed, reduced in scale, or compromised in quality. |
| Schools will prioritize bullying prevention and incorporate it into their daily operations. | Such an assumption is based on the hope that school administration and leadership will see bullying prevention as a central aspect of their educational goal, not an intermittent initiative or outside requirement. | - Schools may treat anti-bullying efforts as optional or peripheral. - Activities could become infrequent, symbolic, or disconnected from student life. - Without leadership prioritization, student and teacher engagement may diminish over time. |
| Students and parents will be open to participating in awareness campaigns and support programs. | This assumption reconizes the central position that parents and students have in the success of anti-bullying initiatives. For the logic model to be effective, it assumes that these two groups of stakeholders will participate, learn, and partner with schools in anti-bullying activities. | Low participation could lead to ineffective outreach, limited impact, and missed opportunities for prevention.  Parents may mistrust or misunderstand the school's efforts, especially if not properly engaged or informed. |

**Table 7: Further Assumptions of the Logic Model**

| **External Factors:** | **Why Important** | **Implications for Implementation:** |
| --- | --- | --- |
| Cultural Attitudes: Prevalent societal attitudes towards bullying and behavior management may impact the acceptance and effectiveness of the model. The UAE’s focus on promoting tolerance and respect provides a favorable cultural backdrop, but shifts in societal norms or resistance to change could pose challenges. | Cultural orientations are potent external factors which may help or obstruct the promotion of anti-bullying strategies. They lie outside immediate control of the program but have to be noted and tracked over its implementation. | •cultural sensitivity - is fundamental. programs should be sensitive and responsive to local norms, using gentle nudges toward change rather than trying to impose external views. • Regular consultations with parents, community leaders, and teachers allow resistance to decrease and trust to build. • The use of positive messaging regarding respect, dignity, and shared values can reconcile anti-bullying objectives with the local customs and religious principles. • Regular cultural context checks as a component of monitoring and evaluation program ensures that the model remains attuned to: - Changeful society attitudes - Emerging challenges in student behavior - Community feedback and expectations |
| Government Policies: Changes in national or local government policies regarding school safety, student rights, or bullying prevention could affect the implementation of the model. | Government policies have a central role in determining the model and the scope of anti-bullying programs. School safety and child protection decisions at the national and local levels can either support the model or constrain it. Policy changes—whether school regulations, safety standards, or child protection legislation—hence need to be monitored closely to ensure that they are aligned with the model objectives. | • Be Informed: Schools and stakeholders should be kept current on updates to government policy on:   - School safety regulations - Student well-being legislation - National anti-bullying strategies   • Adjust Flexibly: The model will need to be flexible and sensitive to changes in policy, such that it can adapt to emerging regulations without sacrificing its central purposes of safety and inclusion. • Collaboration and Advocacy: Strong partnerships between schools and local education authorities must be built in order to:   - Make an impact on policy in favor of bullying prevention. - Guarantee sustained support and funding for anti-bullying efforts. |
| Economic Constraints: Economic challenges or budget cuts could limit the resources available for implementing the intervention. | Economic considerations, for example, budget limitations or financial constraints, are key external factors that can play a significant role in determining the scope, sustainability, and effectiveness of anti-bullying programs. In the event of budget cuts or constrained resources for schools, it might hinder their capacity to undertake major activities such as training, campaigns, support programs, and infrastructure development in reporting and monitoring bullying. | To minimize risks related to economic limitations, the following can be considered:   1. Resource-Effective Allocation: • Put emphasis on core activities like teacher training, awareness campaigns, and establishing reporting systems, that are the strength of the anti-bullying framework. • Implement cost-effective measures, such as peer-based initiatives and online resources (e.g., online training, online surveys, virtual support groups). 2. Sources of Alternative Funding: • Seek extraneous sources of funding, such as:  - Educational program grants by the government. - Partnershipswith community organizations or NGOs dedicated to child protection. - Corporate sponsorships for specific campaigns or activities.  1. Community and Volunteer Engagement: • Utilize community volunteers, i.e., parents, students, and local groups, to assist in conducting activities at little to no cost. • In-kind donations (venues for workshops, gratis materials) by local organizations or community leaders may assist in defraying program costs. 2. Working Together with Other Schools: • Schools can work together on joint initiatives, sharing the expenses and resources for training programs, awareness events, and peer support activities. |
| Technological Influences: The rise of cyberbullying and the increasing use of technology in students' daily lives could necessitate additional strategies beyond traditional bullying prevention methods. | As technology becomes more integrated into daily life, bullying has also grown beyond physical and verbal communication. Cyberbullying—bullying that takes place through electronic means like social media, messaging applications, or video games—is now one of the most common types of bullying among students. Since students spend a lot of time online, it is important that anti-bullying programs evolve to deal with the specific challenges of electronic communication. These technological influences can bring new threats, as well as new opportunities for bullying prevention initiatives. | 1. Revise the Anti-Bullying Curriculum • Schools must incorporate digital citizenship into anti-bullying initiatives: no Instruct students in the responsible use of technology, emphasizing empathy, respect, and communication online. no Educate students about preventing cyberbullying, including how to maintain their privacy and report incidents of bullying online. 2. Cyberbullying Awareness Campaigns •Awareness campaigns must be revised to emphasize the risks of cyberbullying and how it is different from face-to-face bullying. This might include:  - digital literacy training on recognizing and responding to cyberbullying. - Interactive online workshops through which students may learn about the consequences of their digital trail and online actions.  1. Parent and Teacher Training • Teachers and parents need to be trained in recognizing the danger of cyberbullying and assisting students experiencing it:  - Parent workshops in monitoring children's online activity and having constructive talks about the potential risks of digital interactions. - Instructor professional training in identifying symptoms of cyberbullying and giving proper responses.  1. Partnering with Tech Providers •Tech firms can be partnered with, or existing systems used, to develop materials to assist students to report bullying and seek help on the internet: EducaTional tools or systems for reporting cyberbullying can be assisted in developing through platforms. 2. Data Privacy and Protection • With the increase in technology use, schools need to have robust policies in place for protecting data and student privacy:  - Ensure that all online platforms used for reporting or educational purposes are secure and that they protect student data. - Create guidelines for preserving student privacy yet permitting effective monitoring and intervention. |
